# Hybrid Probabilistic Information Set and Multi-Criteria Group Decision-Making Approach: A Case Study to Evaluate Urban Flood Resilience

**DOI:** 10.3390/e28060587

**Published:** 2026-05-25

**Authors:** Xiang He, Yanzhu Hu, Yingjian Wang, Zhen Liang, Binbin Xu

**Affiliations:** 1 School of Intelligent Engineering and Automation, Beijing University of Posts and Telecommunications, Beijing 100876, China; hexiang6@bupt.edu.cn (X.H.); wangyingjian@bupt.edu.cn (Y.W.); liangzhen@bupt.edu.cn (Z.L.); xubinbin@bupt.edu.cn (B.X.); 2 Key Laboratory of IoT Monitoring, Ministry of Emergency Management, Beijing University of Posts and Telecommunications, Beijing 100876, China

**Keywords:** urban flood resilience, hybrid probabilistic information set, cloud model, uncertainty preservation, multi-criteria group decision-making

## Abstract

In recent years, multi-criteria group decision-making (MCGDM) methods have attracted widespread attention in the academic community. However, most existing MCGDM approaches suffer from limitations in decision-makers’ expressive capacity and the loss of uncertain information. To address these issues, this study proposes a novel multi-criteria group decision-making (MCGDM) framework. First, we developed an evaluation information representation method called the hybrid probabilistic information set (HPIS), which allows DMs to fully express their opinions based on individual cognition using the most suitable form of representation. Second, the criteria importance through inter-criteria correlation (CRITIC) and the combined compromise solution (CoCoSo) methods are extended into the cloud model environment, ensuring that the rich uncertainty information is fully preserved and transmitted throughout the entire evaluation process. Finally, we apply the proposed MCGDM framework to a practical case study evaluating urban flood resilience within an urban agglomeration, to identify its vulnerable components. The results indicate that Baoding, Zhangjiakou, and Chengde are identified as the most vulnerable cities, necessitating immediate and targeted measures to bolster their flood defense capabilities. At the same time, decision-makers can select both qualitative and quantitative comments simultaneously and carry uncertainty information throughout the entire calculation process. Furthermore, the sensitivity and comparative analyses demonstrate the robustness and practical utility of the proposed method under the tested scenarios.

## 1. Introduction

Multi-criteria group decision-making (MCGDM) has emerged as a fundamental tool through group decision-making, structured decomposition, and multi-criteria integration [1,2,3]. However, most scholars have focused on the structural decomposition, the construction of comprehensive evaluation criteria systems, and multi-criteria integration [4,5,6,7,8,9,10,11,12,13]. Few studies have focused on the scope of evaluative information selection in group decision-making and the full-process management of complex uncertainties. Obviously, the range of available evaluation information directly affects the decision-makers’ freedom of judgment and the reliability of information sources, while the full-process management of uncertainty determines the rigor and consistency of the evaluation process. Together, these two aspects ultimately determine the accuracy and credibility of the MCGDM results. To fill this gap, this paper focuses on extending the selection domain of evaluation information and managing complex uncertainties throughout the entire evaluation process. In terms of evaluation information expression, although previous linguistic term sets [14,15,16,17,18,19,20,21,22,23] have greatly enriched the options available to decision-makers (DMs), they still exhibit dual structural limitations and fail to fully meet the evaluation needs of DMs.

In terms of complex uncertainty management, previous methods [24,25,26,27,28,29,30,31,32,33] generally suffer from information loss during the uncertainty handling process, making it difficult to ensure that uncertainty is consistently addressed throughout the entire evaluation process. Based on the above two aspects of research content, the following challenges arise:

(1) Existing linguistic term sets present a dual structural limitation when representing DMs’ judgments: comments (such as “excellent” or “good”) can only provide qualitative information, while probabilities (such as “0.8” or “0.5”) can only offer quantitative data. This overlooks the inherent differences in DMs’ cognitive patterns. The framework of structural limitations forces decision-makers to distort their natural expression habits, which may lead to information distortion or reduced engagement.
(2) Existing urban flood resilience assessment methods suffer from fundamental shortcomings in handling uncertainty. Some methods rely entirely on precise values throughout the process, completely discarding uncertainty and reducing complex systems to a deterministic illusion. Others consider uncertainty only at certain stages, resulting in partial information loss and inconsistencies between different phases of the assessment. Methods that rely solely on fuzziness throughout the entire process preserve only a single dimension of uncertainty (fuzziness) while neglecting other critical aspects, such as randomness, thus still representing a “partial preservation” of uncertainty.

To address the above-mentioned challenges, this paper introduces a novel MCGDM framework under the hybrid probabilistic information set (HPIS) based on the Cloud-CRITIC (C-CRITIC) and Cloud-CoCoSo (C-CoCoSo) methods. On the one hand, this study develops a hybrid probabilistic information set whose core innovation lies in decoupling the expression forms of linguistic terms and probabilities. This allows DMs to freely combine qualitative/quantitative expressions for both the comments and their associated probabilities, fundamentally breaking the expressive constraints of existing methods and greatly expanding the range of choices available to DMs. On the other hand, this study innovatively extends the CRITIC and CoCoSo methods to the cloud model environment, enabling a unified representation of both fuzziness and randomness within the evaluation information. This ensures that the rich uncertainty is preserved and transmitted throughout the entire evaluation process, eliminating the fragmentation and loss of uncertainty that occur in traditional evaluation.

In summary, the main contributions of this paper are as follows:

(1) A novel evaluation information representation method, HPIS, is proposed to expand the domain of expression available to DMs. Conversion and normalization procedures, operational rules, and comparative methodologies are also defined.

(2) The improved CRITIC method is extended to the cloud model environment, enabling a unified encoding of both fuzziness and randomness within the evaluation information. The inherent uncertainty contained in the evaluation information is incorporated into the weight calculation process, resulting in a cloud weight triplet together with contrast intensity and conflict. This approach is well-suited for weight determination in complex, uncertain environments.

(3) The CoCoSo techniques are extended to the cloud model environment, where the Bhattacharyya distance is employed to precisely quantify the similarity between cloud models. This enhances the discriminative capability among the cloud models and improves the ranking performance for evaluating the flood resilience of alternative cities.

(4) A novel MCGDM framework is developed using HPIS based on the C-CRITIC and C-CoCoSo methods and is applied for the first time to the evaluation of urban flood resilience. This framework not only yields effective evaluation results but also ensures that complex uncertainty is consistently integrated throughout the entire decision-making process.

The remaining portions of this work are organized as follows: Section 2 provides an overview of the relevant literature. Section 3 defines HPIS along with its standardization process, operational rules, and comparison method between HPICSs. Section 4 presents an innovative MCGDM framework. Section 5 takes the “Beijing Peripheral Urban Agglomeration” as a case study. Subsequently, Section 6 presents a detailed analysis and discussion, including result analysis, sensitivity analysis, comparative analysis with existing methods, and more. Finally, Section 7 summarizes the research findings.

## 2. Literature Review

To address the above challenges, the following will present a review of relevant research on multi-criteria group decision-making (MCGDM) issues, divided into three sections. Section 2.1 provides a review of evaluation information expression methodologies. Section 2.2 presents the methods for determining the weights of DMs and criteria, while Section 2.3 describes the approach for ranking the alternative cities.

### 2.1. Evaluation Information Expression Methodologies

Zadeh (1975) [14] proposed the concept of linguistic variables, which offered a reasoning paradigm capable of handling imprecise information. This framework provides a more realistic representation of human reasoning compared to traditional binary logic. Based on linguistic variables, Herrera et al. (1995) [15] proposed one of the most widely used additive linguistic term sets, which allows DMs to express their opinions about alternatives using linguistic expressions. Due to the complexity and uncertainty associated with real-world issues, various extended linguistic term sets have been established, including the uncertain linguistic term set (ULTS) (Xu, 2004) [16], the probabilistic linguistic term set (PLTS) (Pang et al., 2016) [17], and the double hierarchy linguistic term set (DHLTS) (Gou et al., 2017) [18].

**Figure 1:**
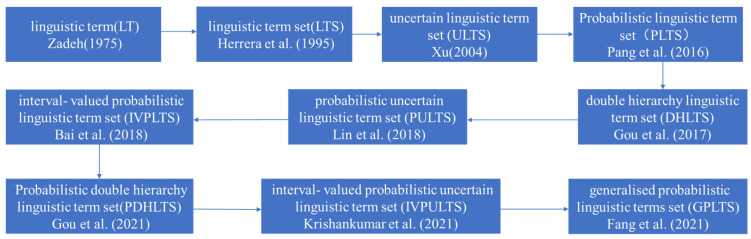
Extensions of the linguistic term set [14,15,16,17,18,19,20,21,22,23].

Figure 1 provides a historical overview of the recent extensions of linguistic term sets.

**Figure 2:**
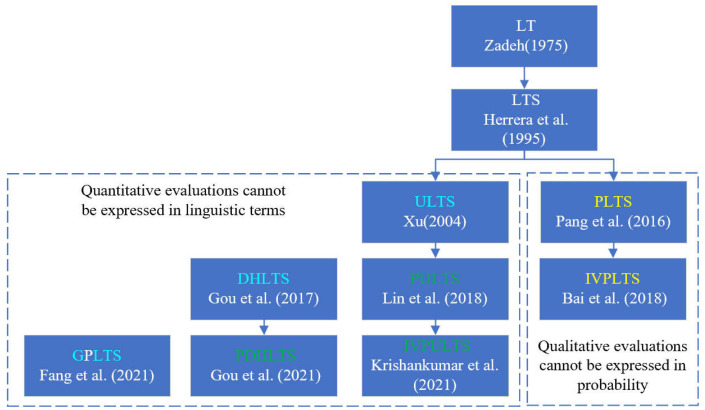
The classification of the extension of linguistic term sets [14,15,16,17,18,19,20,21,22,23].

A categorical analysis of the extended linguistic term sets in Figure 1 is presented in Figure 2. The dark blue section represents linguistic term-based extensions, where Xu (2004) [16] proposed uncertain linguistic term sets (ULTSs) capable of expressing interval-valued linguistic variables. Gou et al. (2017) [18] developed double hierarchy linguistic term sets (DHLTSs) where the secondary LTS provides detailed linguistic supplements or modifiers for each term in the primary LTS. Fang et al. (2021) [23] proposed the generalized probabilistic linguistic term set (GPLTS), which allows the simultaneous expression of single linguistic terms, multiple linguistic terms, and interval linguistic terms. Although these linguistic term sets offer diverse expressive options, they remain limited to qualitative information selection, lacking quantitative evaluation capabilities. The yellow section denotes probability-based extensions. According to Pang et al. (2016) [17], DMs use probability distributions to describe their degree of liking in probabilistic linguistic term sets (PLTS). Bai et al. (2018) [20] subsequently developed interval-valued probabilistic linguistic term sets (IVPLTSs), enabling interval probability expressions to preserve uncertainty in probability estimations. However, these probabilistic approaches restrict probability expressions to quantitative formats, failing to accommodate DMs needing qualitative probability representations. The green section shows hybrid approaches combining linguistic and probabilistic features, including probabilistic uncertain linguistic term sets (PULTSs) (Lin et al., 2018) [19], probabilistic double hierarchy linguistic term sets (PDHLTSs) (Gou et al., 2021) [21], and interval-valued PULTS (IVPULTSs) [22]. For instance, Krishankumar et al. (2021) [22] integrated PULTSs and IVPLTSs into IVPULTSs, allowing simultaneous selection of interval probabilities and interval linguistic terms for uncertainty expression.

Despite enabling separate uncertainty expressions for both probabilities and linguistic terms, these methods still face dual structural limitations: linguistic terms lack quantitative options while probabilities lack qualitative options, inadequate for the complex hybrid-information requirements in MCGDM. To overcome the above limitations, we propose a novel HPIS that allows DMs to freely combine qualitative/quantitative expressions for both the comments and their associated probabilities. Cloud model is employed to uniformly represent this hybrid information, with defined operational rules and comparison methods, fundamentally breaking the expressive constraints of existing methods and greatly expanding the range of choices available to DMs.

### 2.2. Weight Determination Methodologies for Both DMs and Criteria

In multi-criteria decision-making (MCDM) situations, the criteria importance through the inter-criteria correlation (CRITIC) method is frequently employed to determine objective weights. It integrates both the contrast intensity of criteria and the conflict among criteria [34]. The CRITIC technique has been widely used in a variety of fields, including the social sciences, business, and engineering, amongst others [26,35,36,37]. However, in complex multi-criteria group decision-making (MCGDM) problems, abundant uncertainty often exists. The traditional CRITIC method is incapable of capturing the uncertainty embedded in criteria, thereby limiting the reliability of the computed weights. Recently, the CRITIC method has been extended to environments such as intuitionistic, Pythagorean, and interval-valued intuitionistic fuzzy numbers [38]. However, fuzzy sets primarily handle fuzziness, while real-world complex systems often involve a combination of fuzziness, randomness, and other forms of uncertainty. This paper proposes a Cloud-CRITIC (C-CRITIC) method, which extends the traditional CRITIC approach into the cloud model environment. The cloud model is capable of simultaneously representing both fuzziness and randomness, allowing for more comprehensive modeling of criteria. In addition to incorporating contrast intensity and inter-criteria conflict, the C-CRITIC method also integrates the uncertainty of each criterion into the weighting process, resulting in a cloud weight triplet. A lower level of uncertainty indicates a higher degree of confidence in the DMs’ expressed opinions and should therefore be assigned a higher weight. This further enhances the adaptability of the CRITIC method in complex, uncertain environments.

Moreover, as some of the criteria information in MCGDM problems is derived from subjective evaluation by DMs, the conceptual framework of the C-CRITIC method can also be applied to determine DMs’ weights. Thus, this study employs the C-CRITIC method under the cloud model framework to determine both criterion and decision-maker weights.

### 2.3. Alternative City Ranking Methodologies

Regarding alternative city ranking, methods such as vlsekriterijumska optimizacija i kompromisno resenje (VIKOR), technique for order of preference by similarity to ideal solution (TOPSIS), and weighted aggregated sum product assessment (WASPAS), may produce markedly different outcomes when the distributions of criterion weights are altered [30,39]. This indicates reliability and robustness deficiencies in the derived solutions [40]. To address these limitations, Ref. [41] developed the combined compromise solution (CoCoSo) method, integrating simple additive weighting (SAW) and exponentially weighted product (EWP) approaches. The method employs three distinct aggregation strategies to synthesize evaluation information across attributes, ultimately ranking alternative cities through computed utility values [42]. CoCoSo emerges as a preferred ranking method due to its enhanced decision accuracy, absence of rank reversal phenomena, avoidance of division-by-zero or anti-logarithm issues, and superior discrimination capability among alternative cities [35,43]. While subsequent extensions have adapted CoCoSo to various uncertain environments [40,44,45,46], most remain limited to fuzzy set environments. Recognizing fuzzy sets’ constraints in handling comprehensive uncertainty, this paper extends CoCoSo to cloud models, proposing a Cloud-based CoCoSo (C-CoCoSo) approach. The Bhattacharyya distance is employed to calculate the distance measure between cloud models. By assessing the overlap between cloud models, the Bhattacharyya distance determines the similarity between two probability distributions, greatly enhancing the ability to distinguish between cloud models and providing a distance measure that captures distributional differences. Therefore, the C-CoCoSo method provides an effective solution for complex multi-attribute group decision-making problems under uncertain environments.

## 3. Hybrid Probabilistic Information Set (HPIS)

To overcome the limitations of existing linguistic term sets and their extensions, this section proposes a novel concept called the HPIS and provides detailed definitions, normalization procedures, and basic operational rules.

### 3.1. Definition

**Figure 3:**
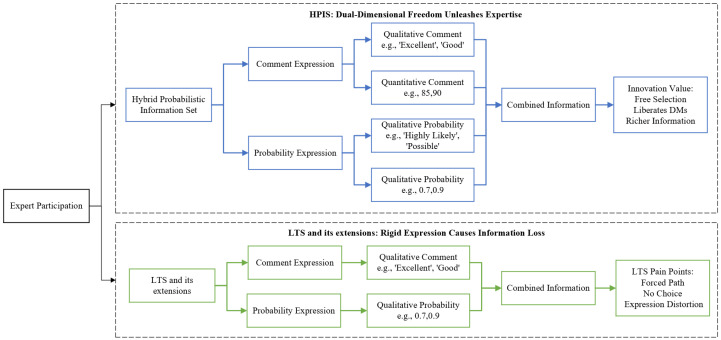
Comparison between the LTS (and its extensions) and HPIS.

Existing linguistic term sets and their extensions have dual structural limitations. For example, when giving comments on the indicators, decision-makers (DMs) can only provide qualitative comments such as “Excellent” and “Good”, but cannot provide quantitative comments such as “85” and “90”. However, DMs in fields such as mathematics or statistics are more sensitive to numbers and prefer to give quantitative comments, while DMs in linguistics or literature are more accustomed to making qualitative comments using language. Furthermore, DMs in interdisciplinary fields may combine qualitative and quantitative information for evaluation. A detailed comparison with HPIS is shown in Figure 3.

#### 3.1.1. Information Set (IS)

To enable DMs to express their opinions on alternative cities using either quantitative or qualitative information according to their individual cognition, this paper proposes a new concept of IS, which is a set containing *K* types of evaluation information, where the evaluation information can be precise values, interval values, linguistic terms, linguistic expressions, etc. The definition is shown in *Equation (1)*:

(1) $$S=\left\{S^{\left(1\right)},S^{\left(2\right)},\ldots ,S^{\left(k\right)}|k=1,2,\ldots ,K\right\}$$

$S^{\left(k\right)}$ represents a possible piece of information within the information set, which may include both quantitative and qualitative information. $K\in N^{+}$ denotes the number of information types contained in the information set S.

**Example** **1** *. Let *S *be an information set. When *K=4* it contains four types of information: exact values, interval values, linguistic terms, and linguistic expressions. The definition is shown in Equation (2).*

(2) $$S=\{S^{\left(1\right)},S^{\left(2\right)},S^{\left(3\right)},S^{\left(4\right)}|S^{\left(1\right)}\in [0,10],S^{\left(2\right)}\in IV,S^{\left(3\right)}\in L,S^{\left(4\right)}\in LE_{c}\}$$

*where* $IV=\left\{[S_{l}^{\left(2\right)},S_{r}^{\left(2\right)}]|S_{l}^{\left(2\right)},S_{r}^{\left(2\right)}\in [0,10],S_{l}^{\left(2\right)}\leq S_{r}^{\left(2\right)}\right\}$ *, the linguistic term set L is as follows:*

$$\begin{array}{l} L=\{L_{0}=\mathrm{none},L_{1}=\mathrm{very}\;\mathrm{low},L_{2}=\mathrm{low},L_{3}=\mathrm{slightly}\;\mathrm{low},L_{4}=\mathrm{medium},L_{5}=\mathrm{slightly}\;\mathrm{high},\\ L_{6}=\mathrm{high},L_{7}=\mathrm{very}\;\mathrm{high},L_{8}=\mathrm{maximum}\} \end{array}$$

*LE_c_* is obtained through *G_H_*, the details of which are provided in Appendix A. Due to limitations on manuscript length, all preliminaries—including definitions, formulas, and related content—are relegated to Appendix A.

To simplify the description, all subsequent defined information sets will contain exact values, interval values, linguistic terms, and linguistic expressions.

#### 3.1.2. HPIS

To represent the degree to which an evaluation object belongs to a specific evaluation information item, the HPIS is proposed, in which the probability can incorporate both qualitative and quantitative information. The definition is shown in *Definition 1*:

**Definition** **1.** *Let *$S_{l}$ *and* $S_{p}$ *be IS, then the HPIS can be defined in Equation (3):*

(3) $$I\left(p\right)=\left\{I^{\left(k\right)}\left(p^{\left(k\right)}\right)|I^{\left(k\right)}\in S_{l},p^{\left(k\right)}\in S_{p},k=1,2,\ldots ,\#I\left(p\right)\right\}$$

 *where* $I^{\left(k\right)}$ *represents the evaluation information, and* $p^{\left(k\right)}$ *represents the probability, both of which can be expressed through the IS.* $\#I\left(p\right)$ *represents the number of hybrid information in the HPIS* $I\left(p\right)$. $S_{l}$ *and* $S_{p}$ *are expressed by Equations (4) and (5).*

(4) $$S_{l}=\{S^{\left(1\right)},S^{\left(2\right)},S^{\left(3\right)},S^{\left(4\right)}|S^{\left(1\right)}\in [0,10],S^{\left(2\right)}\in IV,S^{\left(3\right)}\in L,S^{\left(4\right)}\in LE_{c}\}$$

(5) $$S_{p}=\{S^{\left(1\right)},S^{\left(2\right)},S^{\left(3\right)},S^{\left(4\right)}|S^{\left(1\right)}\in [0,1],S^{\left(2\right)}\in IV,S^{\left(3\right)}\in L_{p},S^{\left(4\right)}\in LE_{p}\}$$

where *L*_p_ is as follows:

$$\begin{array}{l} L_{p}=\{L_{p_{0}}=\mathrm{almost}\;\mathrm{impossible},L_{p_{1}}=\mathrm{highly}\;\mathrm{unlikely},L_{p_{2}}=\mathrm{unlikely},L_{p_{3}}=\mathrm{somewhat}\;\mathrm{unlikely},\\ L_{p_{4}}=\mathrm{possible},L_{p_{5}}=\mathrm{somewhat}\;\mathrm{likely},L_{p_{6}}=\mathrm{likely},L_{p_{7}}=\mathrm{highly}\;\mathrm{likely},L_{p_{8}}=\mathrm{almost}\;\mathrm{certain}\} \end{array}$$

Replace “comment information” with “probabilistic information” in *G_H_*, then determine *LE_p_* according to the modified definition.

### 3.2. Conversion and Normalization

Since the HPIS contains hybrid information in different forms, such as exact values, interval values, linguistic terms, and linguistic expressions, it is necessary to unify the modeling of this hybrid information and ensure that uncertainty is preserved and transmitted in subsequent calculations. Therefore, hybrid information is all uniformly converted into a normal cloud model (NCM). The definition of the NCM and its operational rules can be found in Appendix A.1.

For example, let $S_{lN}$ and $S_{pN}$ be IS, and $I\left(p\right)$ be an HPIS, which is uniformly converted into NCM according to the conversion rule in Appendix A.1.3.

(6) $$S_{lN}=\{f_{E}\left(S^{\left(1\right)}\right),f_{IV}\left(S^{\left(2\right)}\right),f_{LT}\left(S^{\left(3\right)}\right),f_{LE_{c}}\left(S^{\left(4\right)}\right)|S^{\left(1\right)}\in [0,10],S^{\left(2\right)}\in IV,S^{\left(3\right)}\in L,S^{\left(4\right)}\in LE_{c}\}$$

(7) $$S_{pN}=\{f_{E}\left(S^{\left(1\right)}\right),f_{IV}\left(S^{\left(2\right)}\right),f_{LT}\left(S^{\left(3\right)}\right),f_{LE_{p}}\left(S^{\left(4\right)}\right)|S^{\left(1\right)}\in [0,1],S^{\left(2\right)}\in IV,S^{\left(3\right)}\in L_{p},S^{\left(4\right)}\in LE_{p}\}$$

Then, $I\left(p\right)$ is converted into a Hybrid Probability Information Cloud Set (HPICS):

(8) $$I\left(p\right)=\left\{I^{\left(k\right)}\left(p^{\left(k\right)}\right)|I^{\left(k\right)}\in S_{lN},p^{\left(k\right)}\in S_{pN},k=1,2,\ldots ,\#I\left(p\right)\right\}$$

The sum of all probabilities in a single information set is normalized to 1.

(9) $${\dot{p}}^{\left(k\right)}=\frac{p^{\left(k\right)}}{\sum_{k=1}^{\#I(p)}p^{\left(k\right)}}$$

For convenience, the normalized probabilities will be denoted as $p^{\left(k\right)}$ in the following.

### 3.3. Operational Rules of HPICS

**Definition** **2.** *Let* $I_{1}\left(p\right)=\left\{I_{1}^{\left(k\right)}\left(p_{1}^{\left(k\right)}\right)|I_{1}^{\left(k\right)}\in S_{lN},p_{1}^{\left(k\right)}\in S_{pN},k=1,2,\ldots ,\#I_{1}\left(p\right)\right\}$, $I_{2}\left(p\right)=\left\{I_{2}^{\left(k\right)}\left(p_{2}^{\left(k\right)}\right)|I_{2}^{\left(k\right)}\in S_{lN},p_{2}^{\left(k\right)}\in S_{pN},k=1,2,\ldots ,\#I_{2}\left(p\right)\right\}$ *and* $I_{3}\left(p\right)=\left\{I_{3}^{\left(k\right)}\left(p_{3}^{\left(k\right)}\right)|I_{3}^{\left(k\right)}\in S_{lN},p_{3}^{\left(k\right)}\in S_{pN},k=1,2,\ldots ,\#I_{3}\left(p\right)\right\}$ *are three HPICSs,* $\lambda ,\lambda_{1},\lambda_{2}>0$*, then:*

$$I_{1}\left(p\right)⊕I_{2}\left(p\right)=\sum_{k=1}^{\#I_{1}\left(p\right)}\left(I_{1}^{\left(k\right)}\times p_{1}^{\left(k\right)}\right)+\sum_{k=1}^{\#I_{2}\left(p\right)}\left(I_{2}^{\left(k\right)}\times p_{2}^{\left(k\right)}\right)$$

$$I_{1}\left(p\right)⊖I_{2}\left(p\right)=\sum_{k=1}^{\#I_{1}\left(p\right)}\left(I_{1}^{\left(k\right)}\times p_{1}^{\left(k\right)}\right)-\sum_{k=1}^{\#I_{2}\left(p\right)}\left(I_{2}^{\left(k\right)}\times p_{2}^{\left(k\right)}\right)$$

$$I_{1}\left(p\right)⊗I_{2}\left(p\right)=\sum_{k=1}^{\#I_{1}\left(p\right)}\left(I_{1}^{\left(k\right)}\times p_{1}^{\left(k\right)}\right)\times \sum_{k=1}^{\#I_{2}\left(p\right)}\left(I_{2}^{\left(k\right)}\times p_{2}^{\left(k\right)}\right)$$

*where HPICS is the representation obtained after converting the evaluation information and its probabilities in HPIS into cloud models using the conversion and normalization method described in Section 3.2. The addition, subtraction, and multiplication of HPICS are defined here, and the relevant theorems along with their proof processes are provided later.*

**Theorem** **1.** Under the condition of Definition 2, the following relevant theorems concerning addition, subtraction, and multiplication of HPIS are provided.

$$I_{1}\left(p\right)⊕I_{2}\left(p\right)=I_{2}\left(p\right)⊕I_{1}\left(p\right)$$

$$\left(I_{1}\left(p\right)⊕I_{2}\left(p\right)\right)⊕I_{3}\left(p\right)=I_{1}\left(p\right)⊕\left(I_{2}\left(p\right)⊕I_{3}\left(p\right)\right)$$

$$\lambda \left(I_{1}\left(p\right)⊕I_{2}\left(p\right)\right)=\lambda I_{1}\left(p\right)⊕\lambda I_{2}\left(p\right)$$

$$\left(\lambda_{1}+\lambda_{2}\right)I_{1}\left(p\right)=\lambda_{1}I_{1}\left(p\right)⊕\lambda_{2}I_{1}\left(p\right)$$

$$I_{1}\left(p\right)⊖I_{2}\left(p\right)=0⊖I_{1}\left(p\right)⊕I_{2}\left(p\right)$$

$$\left(I_{1}\left(p\right)⊖I_{2}\left(p\right)\right)⊖I_{3}\left(p\right)=I_{1}\left(p\right)⊖\left(I_{2}\left(p\right)⊕I_{3}\left(p\right)\right)$$

$$\lambda \left(I_{1}\left(p\right)⊖I_{2}\left(p\right)\right)=\lambda I_{1}\left(p\right)⊖\lambda I_{2}\left(p\right)$$

$$\left(\lambda_{1}-\lambda_{2}\right)I_{1}\left(p\right)=\lambda_{1}I_{1}\left(p\right)⊖\lambda_{2}I_{1}\left(p\right)$$

$$I_{1}\left(p\right)⊗I_{2}\left(p\right)=I_{2}\left(p\right)⊗I_{1}\left(p\right)$$

$$\left(I_{1}\left(p\right)⊗I_{2}\left(p\right)\right)⊗I_{3}\left(p\right)=I_{1}\left(p\right)⊗\left(I_{2}\left(p\right)⊗I_{3}\left(p\right)\right)$$

$${\left(I_{1}\left(p\right)⊗I_{2}\left(p\right)\right)}^{\lambda }={\left(I_{1}\left(p\right)\right)}^{\lambda }⊗{\left(I_{2}\left(p\right)\right)}^{\lambda }$$

$$I_{1}{\left(p\right)}^{\left(\lambda_{1}+\lambda_{2}\right)}={\left(I_{1}\left(p\right)\right)}^{\left(\lambda_{1}\right)}⊗{\left(I_{1}\left(p\right)\right)}^{\left(\lambda_{2}\right)}$$

The following key theorems will be proven, while others can be inferred in a similar manner and are therefore omitted for brevity.

**Proof.**  The subsequent derivations follow a unified procedure. First, the HPIS is expanded into a cloud model based on Definition 2. Second, the arithmetic operations (addition, subtraction, multiplication, division, etc.) of cloud models, as given in Appendix A.1, are employed to complete the derivations.

$$\begin{array}{cc} I_{1}\left(p\right)⊕I_{2}\left(p\right) & =\sum_{k=1}^{\#I_{1}\left(p\right)}\left(I_{1}^{\left(k\right)}\times p_{1}^{\left(k\right)}\right)+\sum_{k=1}^{\#I_{2}\left(p\right)}\left(I_{2}^{\left(k\right)}\times p_{2}^{\left(k\right)}\right)=\sum_{k=1}^{\#I_{2}\left(p\right)}\left(I_{2}^{\left(k\right)}\times p_{2}^{\left(k\right)}\right)+\sum_{k=1}^{\#I_{1}\left(p\right)}\left(I_{1}^{\left(k\right)}\times p_{1}^{\left(k\right)}\right)\\ & =I_{2}\left(p\right)⊕I_{1}\left(p\right) \end{array}$$

$$\begin{array}{cc} \lambda \left(I_{1}\left(p\right)⊕I_{2}\left(p\right)\right) & =\lambda \left(\sum_{k=1}^{\#I_{1}\left(p\right)}\left(I_{1}^{\left(k\right)}\times p_{1}^{\left(k\right)}\right)+\sum_{k=1}^{\#I_{2}\left(p\right)}\left(I_{2}^{\left(k\right)}\times p_{2}^{\left(k\right)}\right)\right)=\lambda \sum_{k=1}^{\#I_{1}\left(p\right)}\left(I_{1}^{\left(k\right)}\times p_{1}^{\left(k\right)}\right)+\lambda \sum_{k=1}^{\#I_{2}\left(p\right)}\left(I_{2}^{\left(k\right)}\times p_{2}^{\left(k\right)}\right)\\ & =\lambda I_{1}\left(p\right)⊕\lambda I_{2}\left(p\right) \end{array}$$

$$\begin{array}{cc} I_{1}\left(p\right)⊖I_{2}\left(p\right) & =\sum_{k=1}^{\#I_{1}\left(p\right)}\left(I_{1}^{\left(k\right)}\times p_{1}^{\left(k\right)}\right)-\sum_{k=1}^{\#I_{2}\left(p\right)}\left(I_{2}^{\left(k\right)}\times p_{2}^{\left(k\right)}\right)=0-\sum_{k=1}^{\#I_{1}\left(p\right)}\left(I_{1}^{\left(k\right)}\times p_{1}^{\left(k\right)}\right)+\sum_{k=1}^{\#I_{2}\left(p\right)}\left(I_{2}^{\left(k\right)}\times p_{2}^{\left(k\right)}\right)\\ & =0⊖I_{1}\left(p\right)⊕I_{2}\left(p\right) \end{array}$$

$$\begin{array}{cc} {\left(I_{1}\left(p\right)⊗I_{2}\left(p\right)\right)}^{\lambda } & ={\left(\sum_{k=1}^{\#I_{1}\left(p\right)}\left(I_{1}^{\left(k\right)}\times p_{1}^{\left(k\right)}\right)\times \sum_{k=1}^{\#I_{2}\left(p\right)}\left(I_{2}^{\left(k\right)}\times p_{2}^{\left(k\right)}\right)\right)}^{\lambda }\\ & ={\left(\sum_{k=1}^{\#I_{1}\left(p\right)}\left(I_{1}^{\left(k\right)}\times p_{1}^{\left(k\right)}\right)\right)}^{\lambda }+{\left(\sum_{k=1}^{\#I_{2}\left(p\right)}\left(I_{2}^{\left(k\right)}\times p_{2}^{\left(k\right)}\right)\right)}^{\lambda }\\ & ={\left(I_{1}\left(p\right)\right)}^{\lambda }⊗{\left(I_{2}\left(p\right)\right)}^{\lambda } \end{array}$$

□

### 3.4. The Comparison Between HPICSs

After we have given the concept of HPICS, we need to put forward a method to compare HPICSs.

**Definition** **3.** *Let* $I_{1}\left(p\right)=\left\{I_{1}^{\left(k\right)}\left(p_{1}^{\left(k\right)}\right)|I_{1}^{\left(k\right)}\in S_{lN},p_{1}^{\left(k\right)}\in S_{pN},k=1,2,\ldots ,\#I_{1}\left(p\right)\right\}$ *and* $I_{2}\left(p\right)=\left\{I_{2}^{\left(k\right)}\left(p_{2}^{\left(k\right)}\right)|I_{2}^{\left(k\right)}\in S_{lN},p_{2}^{\left(k\right)}\in S_{pN},k=1,2,\ldots ,\#I_{2}\left(p\right)\right\}$ *be two HPICSs, and* $\partial_{1}=\sum_{k=1}^{\#I_{1}\left(p\right)}\left(I_{1}^{\left(k\right)}\times p_{1}^{\left(k\right)}\right)$ *and* $\partial_{2}=\sum_{k=1}^{\#I_{2}\left(p\right)}\left(I_{2}^{\left(k\right)}\times p_{2}^{\left(k\right)}\right)$ *. The comparison of the sizes of* $I_{1}\left(p\right)$ *and* $I_{2}\left(p\right)$ *is as follows: *

$$if\;\partial_{1}>\partial_{2},\;then\;I_{1}\left(p\right)>I_{2}\left(p\right)$$

$$if\;\partial_{1}<\partial_{2},\;then\;I_{1}\left(p\right)<I_{2}\left(p\right)$$

$$if\;\partial_{1}=\partial_{2},\;then\;I_{1}\left(p\right)=I_{2}\left(p\right)$$

## 4. An Innovative MCGDM Approach Based on the C-CRITIC and C-CoCoSo Under HPIS

**Figure 4:**
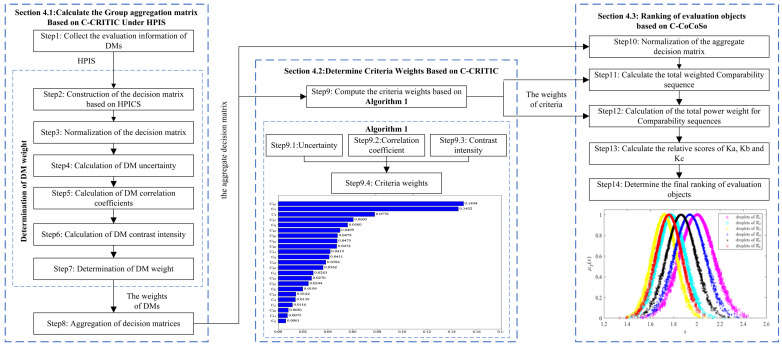
Flowchart of proposed methodology.

The previous section introduced the proposed HPIS, which overcomes the dual limitations of existing linguistic term sets and their extended forms. This section will introduce an innovative multi-criteria group decision-making (MCGDM) method as shown in Figure 4. This method mainly consists of three components: hybrid probabilistic information set (HPIS), the cloud criteria importance through inter-criteria correlation (C-CRITIC), and the cloud combined compromise solution (C-CoCoSo). C-CRITIC and C-CoCoSo operate within a cloud model environment to ensure that uncertainty information is preserved throughout the evaluation process. A detailed description will be given in three parts as follows.

### 4.1. Calculate the Group Aggregation Matrix Based on C-CRITIC Under HPIS

This subsection will introduce the criteria importance through the inter-criteria correlation (CRITIC) method for calculating the weights of decision-makers (DMs). The steps for calculating DMs’ weights based on C-CRITIC are as follows:

Step 1. Collect the evaluation information of DMs.

Suppose there are *m* alternative cities $ℜ=\left\{{ℜ}_{1},{ℜ}_{2},\ldots ,{ℜ}_{m}\right\}$, *n* criteria $C=\left\{C_{1},C_{2},\ldots ,C_{n}\right\}$, and *s* decision-makers $DM=\left\{DM_{1},DM_{2},\ldots ,DM_{s}\right\}$. The evaluation information of the DM is collected in the form of an HPIS.

Step 2. Construction of the decision matrix based on HPICS.

The conversion of evaluation information based on HPIS into Hybrid Probabilistic Information Cloud Set (HPICS) and its normalization is carried out using the method described in Section 3.2. Therefore, the decision matrix is constructed from the evaluation information of the *q*-th decision-maker as shown in *Equation (10)*.

(10) $$D^{q}={\left(\begin{array}{cccc} I_{11}^{q}(p) & I_{12}^{q}(p) & \ldots & I_{1n}^{q}(p)\\ I_{21}^{q}(p) & I_{22}^{q}(p) & \ldots & I_{2n}^{q}(p)\\ \vdots & \vdots & \vdots & \vdots \\ I_{m1}^{q}(p) & I_{m2}^{q}(p) & \ldots & I_{mn}^{q}(p) \end{array}\right)}_{m\times n}$$

where the evaluation information of the *q*-th decision-maker (DM) in $I_{ij}^{q}\left(p\right)=\left\{{I_{ij}}^{\left(k\right)}\left({p_{ij}}^{\left(k\right)}\right)|{I_{ij}}^{\left(k\right)}\in S_{lN},{p_{ij}}^{\left(k\right)}\in S_{pN},k=1,2,\ldots ,\#I_{ij}\left(p\right),i=1,2,\ldots ,m,j=1,2,\ldots ,n\right\}$ is HPICS, and $D^{q}$ is a hybrid probability information cloud decision matrix.

Step 3. Normalization of the decision matrix.

Let the element $I_{ij}^{q}(p)$ in the evaluation information of the *q*-th DM be standardized to an NCM $C_{ij}^{q}=\left(Ex_{ij}^{q},En_{ij}^{q},He_{ij}^{q}\right)$, and the benefit-type and cost-type criteria are standardized through *Equation (11)*.

(11) $$C_{ij}^{q}=\left\{\begin{array}{c} \frac{I_{ij}^{q}(p)⊖I_{j}^{q-}(p)}{I_{j}^{q+}(p)⊖I_{j}^{q-}(p)},\mathrm{Benefit}\\ \frac{I_{j}^{q+}(p)⊖I_{ij}^{q}(p)}{I_{j}^{q+}(p)⊖I_{j}^{q-}(p)},\;\mathrm{Cos}t \end{array}\right.$$

where i=1,2,…,m and j=1,2,…,n are the values, with $I_{j}^{q+}(p)=\overset{m}{\underset{i=1}{\max}}\left\{I_{ij}^{q}(p)\right\}$ as the maximum value and $I_{j}^{q-}(p)=\overset{m}{\underset{i=1}{\min}}\left\{I_{ij}^{q}(p)\right\}$ as the minimum value.

Step 4. Calculation of DM uncertainty

(12) $$\upsilon^{q}=\frac{\sum_{j=1}^{n}\sum_{i=1}^{m}\left(En_{ij}^{q}+3He_{ij}^{q}\right)}{\sum_{q=1}^{s}\sum_{j=1}^{n}\sum_{i=1}^{m}\left(En_{ij}^{q}+3He_{ij}^{q}\right)}$$

where $\upsilon^{q}$ is the uncertainty of the *q*-th DM.

Step 5. Calculation of DM correlation coefficients.

Determine the correlation coefficient $\rho^{qy}$ between each DM using the formula below:

(13) $$\rho^{qy}=\frac{\sum_{j=1}^{n}\sum_{i=1}^{m}BD\left(C_{ij}^{q},{\bar{C}}_{j}^{q}\right)BD\left(C_{ij}^{y},{\bar{C}}_{j}^{y}\right)}{\sqrt{\sum_{j=1}^{n}\sum_{i=1}^{m}{\left(BD\left(C_{ij}^{q},{\bar{C}}_{j}^{q}\right)\right)}^{2}\sum_{j=1}^{n}\sum_{i=1}^{m}{\left(BD\left(C_{ij}^{y},{\bar{C}}_{j}^{y}\right)\right)}^{2}}}$$

where the difference between the criterion and its average value is calculated using the distance measure BD, where ${\bar{C}}_{j}^{q}$ and ${\bar{C}}_{j}^{y}$ represent the average values of the *j*-th criterion in the evaluation information of the *q*-th and *y*-th decision-makers, respectively. This is solved using *Equation (14)*.

(14) $${\bar{C}}_{j}^{q}=\frac{\sum_{i=1}^{m}C_{ij}^{q}}{m},\;\;\;\;\;\;\;\;{\bar{C}}_{j}^{y}=\frac{\sum_{i=1}^{m}C_{ij}^{y}}{m}$$

$\rho^{qy}$ represents the degree of association between the evaluation information of the *q*-th and *y*-th decision-makers.

Step 6. Calculation of DM contrast intensity.

Let $\sigma_{j}^{q}$ represent the sum of the standard deviations of all criteria in the *q*-th decision. It is solved through *Equation (15)*.

(15) $$\sigma^{q}=\sqrt{\frac{\sum_{j=1}^{n}\sum_{i=1}^{m}{\left(BD\left(C_{ij}^{q},{\bar{C}}_{j}^{q}\right)\right)}^{2}}{n\times m}}$$

Step 7. Determination of DM weight.

Utilize *Equation (16)* to determine the index $\eta^{q}$.

(16) $$\eta^{q}=(1-\upsilon^{q})\sigma^{q}\sum_{y=1}^{s}\left(1-\rho^{qy}\right)$$

Use *Equation (17)* to determine the DM weight.

(17) $$w^{q}=\frac{\eta^{q}}{\sum_{q=1}^{s}\eta^{q}}$$

Step 8. Aggregation of decision matrices.

Obtain the aggregated results $C={\left(C_{ij}\right)}_{m\times n}$ of DMs’ evaluation information through arithmetic weighted averaging.

(18) $$C_{ij}=\left(Ex_{ij},En_{ij},He_{ij}\right)=\sum_{q=1}^{s}w^{q}\left(Ex_{ij}^{q},En_{ij}^{q},He_{ij}^{q}\right)$$

### 4.2. Determine Criteria Weights Based on C-CRITIC

This subsection will introduce the C-CRITIC method for calculating the weights of criteria. Since the calculation of DMs’ weights is based on three-dimensional data of criteria, and the calculation of criteria weights is based on two-dimensional data of criteria, the formulas differ. Therefore, the calculation steps for the weights of the criteria need to be introduced separately. The steps for calculating criteria weights based on C-CRITIC are as follows:

Step 9. Determination of criteria weights ($w_{j}$) by Algorithm A1.

### 4.3. Ranking of Alternative Cities Based on C-CoCoSo

This section will introduce the proposed cloud combined compromise solution (C-CoCoSo) algorithm to calculate the ranking results of the alternative cities. The steps are as follows:

Step 10. Normalization of the aggregate decision matrix

(19) $$C_{ij}=\left\{\begin{array}{c} \frac{C_{ij}+C_{j}^{+}-2C_{j}^{-}}{C_{j}^{+}-C_{j}^{-}},\mathrm{Benefit}\\ \frac{2C_{j}^{+}-C_{ij}-C_{j}^{-}}{C_{j}^{+}-C_{j}^{-}},\;\mathrm{Cos}t \end{array}\right.$$

where i=1,2,…,m and j=1,2,…,n, the maximum value $C_{j}^{+}=\overset{m}{\underset{i=1}{\max}}\left\{C_{ij}\right\}$ and the minimum value $C_{j}^{-}=\overset{m}{\underset{i=1}{\min}}\left\{C_{ij}\right\}$. Since *Equation (11)* has already performed standardized preprocessing on the decision matrix, all criteria in this step are calculated as benefit-type criteria. To avoid the occurrence of a denominator being zero (or approaching zero) in subsequent calculations, an improved normalization formula *Equation (19)* is proposed, ensuring that the normalized *Ex* ranges from [1, 2].

Step 11. For each alternative city ${\mathbb{Q}}_{i}$, determine the sum of the weighted comparability sequence.

(20) $${\mathbb{Q}}_{i}=\sum_{j=1}^{n}\left(w_{j}C_{ij}\right),i=1,2,\ldots ,m$$

where $w_{j}$ is weights of criteria and $C_{ij}$ is an element of the aggregated decision matrix.

Step 12. For every alternative city ${\mathbb{N}}_{i}$, determine the total power weight of the comparability sequence.

(21) $${\mathbb{N}}_{i}=\sum_{j=1}^{n}{\left(C_{ij}\right)}^{w_{j}},i=1,2,\ldots ,m$$

Step 13. Calculate the relative scores $K_{ia},K_{ib}$ and $K_{ic}$ of the alternative cities.

Three scoring strategies are used to generate the relative scores of the alternative cities, as shown in the following formulas:

(22) $$K_{ia}=\frac{{\mathbb{Q}}_{i}+{\mathbb{N}}_{i}}{\sum_{i=1}^{m}\left({\mathbb{Q}}_{i}+{\mathbb{N}}_{i}\right)},i=1,2,\ldots ,m$$

(23) $$K_{ib}=\frac{{\mathbb{Q}}_{i}}{\underset{i}{\min}{\mathbb{Q}}_{i}}+\frac{{\mathbb{N}}_{i}}{\underset{i}{\min}{\mathbb{N}}_{i}},i=1,2,\ldots ,m$$

(24) $$K_{ic}=\frac{\xi {\mathbb{Q}}_{i}+(1-\xi ){\mathbb{N}}_{i}}{\xi \underset{i}{\max}\mathbb{Q}+(1-\xi )\underset{i}{\max}\mathbb{N}},i=1,2,\ldots ,m$$

where $K_{ia}$ is the weighted sum method (WSM) and weighted product method (WPM) scores’ arithmetic mean, $K_{ib}$ is the sum of the WSM and WPM scores relative to the optimal, and $K_{ic}$ is the balanced compromise between the WSM and WPM scores. The balance coefficient $\xi \left(0<\xi <1\right)$ reflects the security and adaptability of the CoCoSo algorithm and is generally set to 0.5.

Step 14. Determine the final ranking of alternative cities.

(25) $$K_{i}=\sqrt[{3}]{K_{ia}K_{ib}K_{ic}}+\frac{1}{3}\left(K_{ia}+K_{ib}+K_{ic}\right),i=1,2,\ldots ,m$$

where $K_{i}$ represents the ranking value of the alternative city. The higher the $K_{i}$ value, the higher the ranking of the alternative city.

## 5. Case Study

**Figure 5:**
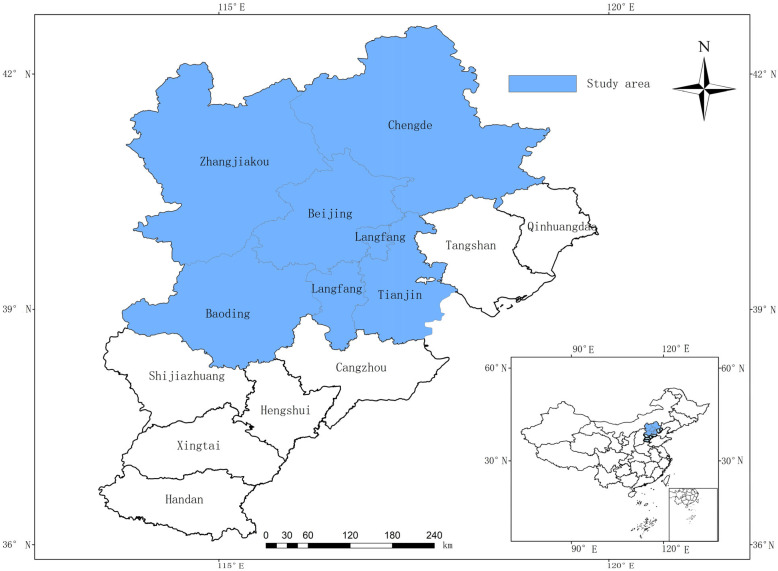
“Beijing Peripheral Urban Agglomeration” study area.

Beijing, Tianjin, and eleven cities at the prefecture level from Hebei province make up the Beijing–Tianjin–Hebei region. The region has a high population density, a lot of economic activity, and valuable assets and infrastructure. Since 1956, heavy rainfall and waterlogging have frequently affected urban safety in the Beijing–Tianjin–Hebei area, especially in 2016, when 9.4785 million people were affected, causing direct economic losses of 55.087 billion yuan [47]. This study selects the “Beijing Peripheral Urban Agglomeration” (BPUA) as the research area, which includes a total of six cities centered around Beijing and adjacent to its boundaries—namely Chengde, Langfang, Tianjin, Baoding, and Zhangjiakou—from the Beijing–Tianjin–Hebei (Jing–Jin–Ji) region, as illustrated in Figure 5. In 2023, the per capita GDP of Beijing was approximately 200,000 yuan and Tianjin’s per capita GDP was around 123,000 yuan, while the other prefecture-level cities had a per capita GDP of less than 70,000 yuan, leading to varying levels of resilience to heavy rainfall and waterlogging in the BPUA. However, cities within the urban cluster are highly interconnected in terms of economy, transportation, resources, and other fields. When one city encounters a flood disaster, it may impact logistics, supply chains, energy, and communication systems across the entire urban cluster. Therefore, identifying the weak links in the flood resilience of the BPUA and the key factors affecting the urban flood resilience is crucial to enhancing the overall flood resilience capacity of the region.

**Figure 6:**
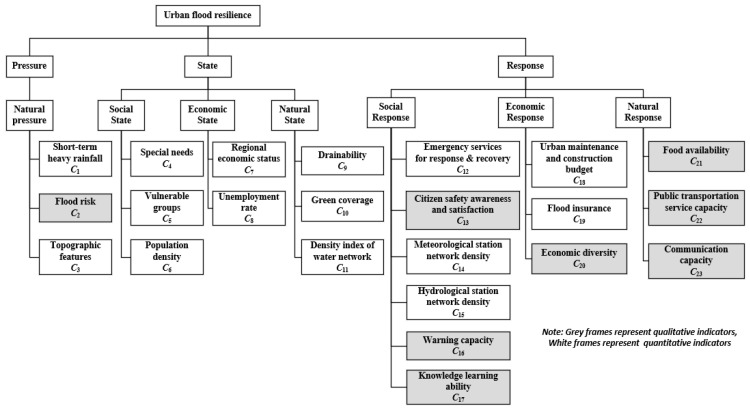
Urban flood resilience evaluation criteria system.

The criteria system, consisting of 23 indicators(*C*_1_–*C*_23_), was constructed based on the urban flood resilience evaluation framework developed by He et al. (2024) [48] under the PSR-SENCE theoretical model, as shown in Figure 6. Four decision-makers (DMs) (denoted as $DM=\left\{DM_{1},DM_{2},DM_{3},DM_{4}\right\}$), each with more than five years of experience in the relevant field, were invited to use HPIS to evaluate the flood resilience of the six cities in the BPUA—Beijing, Chengde, Baoding, Tianjin, Langfang, and Zhangjiakou (collectively referred to as alternative cities $ℜ=\left\{{ℜ}_{1},{ℜ}_{2},{ℜ}_{3},{ℜ}_{4},{ℜ}_{5},{ℜ}_{6}\right\}$)—based on the established criteria system for the year 2022. The information of the decision-makers is shown in Table 1.

The flood resilience ranking of six cities in the study area will be conducted through a three-phase computational process comprising 14 steps, with the overall framework illustrated in Figure 4. Phase I involves using the hybrid probabilistic information set (HPIS) to evaluate the criteria based on DMs’ evaluations, and determining the weights of DMs using the proposed C-CRITIC method, thereby obtaining the group aggregation decision matrix. In Phase II, based on the aggregated decision matrix obtained in Step 1, the criteria weights are calculated using the C-CRITIC method. Phase III employs the cloud model-extended C-CoCoSo method to rank flood resilience across six cities in the BPUA study area.

Step 1. DMs use the HPIS to express evaluation information in the way that is most familiar and comfortable for them. Collect all DMs’ evaluation information. These evaluation results are shown in Table A2.

Step 2. The decision matrix is constructed based on the evaluation information provided by the DMs and then converted and normalized into a hybrid probabilistic information cloud decision matrix $D=\left\{D^{1},D^{2},D^{3},D^{4}\right\}$ using the methods described in Section 3.2, as shown in *Equation (26)*. The remaining elements can be derived similarly.

(26) $$D^{1}={\left(\begin{array}{cccc} I_{1,1}^{1}(p) & I_{1,2}^{1}(p) & \cdots & I_{1,23}^{1}(p)\\ I_{2,1}^{1}(p) & I_{2,2}^{1}(p) & \cdots & I_{2,23}^{1}(p)\\ \vdots & \vdots & \vdots & \vdots \\ I_{6,1}^{1}(p) & I_{6,2}^{1}(p) & \cdots & I_{6,23}^{1}(p) \end{array}\right)}_{6\times 23}$$

where

$$I_{1,1}^{1}(p)=\left\{\left(2.5,0,0\right)\left(0.8577,0.1855,0.006\right),\left(3,0,0\right)\left(0.1227,0.1011,0.003\right),\left(2,0,0\right)\left(0.0196,0.0453,0.002\right)\right\}$$

$$I_{1,2}^{1}(p)=\left\{\left(3.6331,2.4015,0.0981\right)\left(0.9,0,0\right),\left(10,0,0\right)\left(0.1,0,0\right)\right\}$$

$$I_{1,23}^{1}(p)=\left\{\left(9.7750,1.0540,0.0270\right)\left(0.9,0,0\right),\left(10,0.1,0.02\right)\left(0.1,0,0\right)\right\}$$

Two bolded elements in Table A2 are converted into HPICS using the exact value conversion formula in Equation (A10) and the cloud model mapping rule $f_{LT}$.

Step 3. The HPIC decision matrix is normalized using *Equation (11)*, resulting in the normalized decision matrix $\hat{C}=\left\{C^{1},C^{2},C^{3},C^{4}\right\}$, where $C^{1}$ is shown in *Equation (27)*. The rest can be derived similarly.

(27) $$C^{1}={\left(\begin{array}{cccc} C_{1,1}^{1} & C_{1,2}^{1} & \cdots & C_{1,23}^{1}\\ C_{2,1}^{1} & C_{2,2}^{1} & \cdots & C_{2,23}^{1}\\ \vdots & \vdots & \vdots & \vdots \\ C_{6,1}^{1} & C_{6,2}^{1} & \cdots & C_{6,23}^{1} \end{array}\right)}_{6\times 23}$$

where

$$C_{11}^{1}=\left(0,0.5625,0.0180\right),$$

$$C_{12}^{1}=\left(1,0.7553,0.0296\right),$$

$$C_{1,23}^{1}=\left(1,0.6039,0.0234\right).$$

Step 4. Calculate the uncertainty measures for all DMs using *Equation (12)*. The resulting uncertainty matrix is presented below.

(28) υ=[0.1939,0.1927,0.325,0.2884]

Step 5. Compute correlation coefficients for all DMs using *Equations (13)* and *(14)*. The comparison matrix is presented as follows:

(29) $$\rho =\left[\begin{array}{cccc} \begin{array}{c} 1.000\\ 0.3910\\ 0.2403\\ 0.3170 \end{array} & \begin{array}{c} 0.3910\\ 1.000\\ 0.2664\\ 0.3600 \end{array} & \begin{array}{c} 0.2403\\ 0.2664\\ 1.000\\ 0.5036 \end{array} & \begin{array}{c} 0.3170\\ 0.3600\\ 0.5036\\ 1.000 \end{array} \end{array}\right]$$

Step 6–Step 7. Calculate all DMs’ contrast intensity using *Equation (15)*. Compute the index $\eta^{q}$ using *Equation (16)*, and determine decision-maker weights through *Equation (17)*, with results shown in Table 2.

Step 8. Construct the aggregated decision matrix $C={\left(C_{nm}\right)}_{6\times 23}$, with its elements calculated using *Equation (18)*. The specific values are shown in Table A3.

Step 9. The weights of all criteria are calculated using Algorithm A1, as shown in Table A3. Since the method for determining criteria weights is the same as that for calculating DM weights, the calculation process is not repeated.

Step 10. The aggregated decision matrix $\mathbb{C}={\left({\mathbb{C}}_{nm}\right)}_{6\times 23}$ is standardized using *Equation (19)*.

Step 11–Step 12. Using the criteria weights from Table A3: Calculate weighted comparable sequence sums ℚ for all alternative cities using *Equation (27)*. Compute power-weighted comparable sequence sums ℕ through *Equation (28)*. The calculation results are presented below.

$$\mathbb{Q}=\begin{array}{r} {ℜ}_{1}\\ {ℜ}_{2}\\ {ℜ}_{3}\\ {ℜ}_{4}\\ {ℜ}_{5}\\ {ℜ}_{6} \end{array}\left\{\begin{array}{r} \left(1.8286,0.2389,0.0093\right)\\ \left(1.3356,0.1800,0.0070\right)\\ \left(1.1919,0.1582,0.0062\right)\\ \left(1.6882,0.2076,0.0081\right)\\ \left(1.5153,0.1960,0.0076\right)\\ \left(1.2793,0.1701,0.0066\right) \end{array}\right\},\;\mathbb{N}=\begin{array}{r} {ℜ}_{1}\\ {ℜ}_{2}\\ {ℜ}_{3}\\ {ℜ}_{4}\\ {ℜ}_{5}\\ {ℜ}_{6} \end{array}\left\{\begin{array}{r} \left(23.5930,0.5336,0.0203\right)\\ \left(23.2679,0.5741,0.0218\right)\\ \left(23.1533,0.5792,0.0219\right)\\ \left(23.5193,0.5214,0.0197\right)\\ \left(23.4122,0.5355,0.0203\right)\\ \left(23.2348,0.5616,0.0214\right) \end{array}\right\}$$

Step 13. Calculate the relative scores for all alternative cities through three aggregation approaches: ($K_{a}$) *Equation (22)*, (*K_b_*) *Equation (23)*, and ($K_{c}$) *Equation (24)*.

$$K_{a}=\begin{array}{r} {ℜ}_{1}\\ {ℜ}_{2}\\ {ℜ}_{3}\\ {ℜ}_{4}\\ {ℜ}_{5}\\ {ℜ}_{6} \end{array}\left\{\begin{array}{r} \left(0.1706,0.0043,0.0002\right)\\ \left(0.1651,0.0043,0.0002\right)\\ \left(0.1634,0.0043,0.0002\right)\\ \left(0.1692,0.0041,0.0002\right)\\ \left(0.1673,0.0041,0.0002\right)\\ \left(0.1645,0.0042,0.0002\right) \end{array}\right\},K_{b}=\begin{array}{r} {ℜ}_{1}\\ {ℜ}_{2}\\ {ℜ}_{3}\\ {ℜ}_{4}\\ {ℜ}_{5}\\ {ℜ}_{6} \end{array}\left\{\begin{array}{r} \left(2.5532,0.2878,0.0112\right)\\ \left(2.1255,0.2149,0.0083\right)\\ \left(2.0000,0.1910,0.0074\right)\\ \left(2.4322,0.2585,0.0101\right)\\ \left(2.2825,0.2381,0.0093\right)\\ \left(2.0768,0.2046,0.0080\right) \end{array}\right\}$$

$$K_{c}=\begin{array}{r} {ℜ}_{1}\\ {ℜ}_{2}\\ {ℜ}_{3}\\ {ℜ}_{4}\\ {ℜ}_{5}\\ {ℜ}_{6} \end{array}\left\{\begin{array}{r} \left(1.0000,0.0325,0.0012\right)\\ \left(0.9678,0.0325,0.0012\right)\\ \left(0.9577,0.0323,0.0012\right)\\ \left(0.9916,0.0317,0.0012\right)\\ \left(0.9806,0.0318,0.0012\right)\\ \left(0.9643,0.0320,0.0012\right) \end{array}\right\}$$

Step 14. Determine the final ranking of alternative cities for *Equation (25)*.

$$K=\begin{array}{r} {ℜ}_{1}\\ {ℜ}_{2}\\ {ℜ}_{3}\\ {ℜ}_{4}\\ {ℜ}_{5}\\ {ℜ}_{6} \end{array}\left\{\begin{array}{r} \left(1.9993,0.1490,0.0058\right)\\ \left(1.7838,0.1167,0.0045\right)\\ \left(1.7192,0.1056,0.0041\right)\\ \left(1.9393,0.1355,0.0053\right)\\ \left(1.8642,0.1267,0.0049\right)\\ \left(1.7592,0.1118,0.0043\right) \end{array}\right\}$$

## 6. Analysis and Discussion

### 6.1. Result Analysis

**Figure 7:**
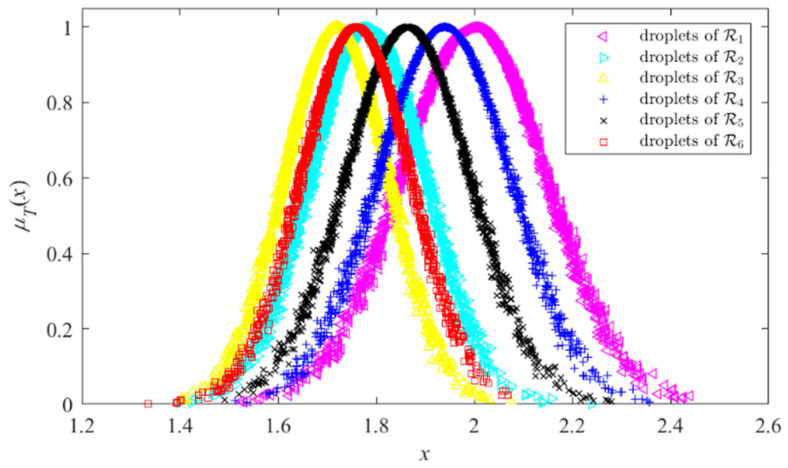
Cloud drop diagrams of each alternative city.

The cloud drop diagrams of each alternative city are shown in Figure 7. By comparing the size of K using Definition A4, it can be concluded that ${ℜ}_{1}>{ℜ}_{4}>{ℜ}_{5}>{ℜ}_{2}>{ℜ}_{6}>{ℜ}_{3}$. The top three cities in terms of flood resilience are Beijing (${ℜ}_{1}$), Tianjin (${ℜ}_{4}$), and Langfang (${ℜ}_{5}$). This is primarily because Beijing, as the capital, benefits from substantial investment in flood control infrastructure, advanced early warning systems, and high emergency response capacity. Tianjin has made significant progress in sponge city construction and drainage system upgrades, while Langfang, located between Beijing and Tianjin, has leveraged regional coordination to improve its resilience. In contrast, Baoding (${ℜ}_{3}$), Zhangjiakou (${ℜ}_{6}$), and Chengde (${ℜ}_{2}$) lag behind due to relatively weaker economic bases, aging drainage facilities, and lower investment in disaster adaptation. The uncertainty represented by UD=En+3He is shown in Table 3 for each alternative city’s results. Clearly, $UD_{{ℜ}_{1}}>UD_{{ℜ}_{4}}>UD_{{ℜ}_{5}}>UD_{{ℜ}_{2}}>UD_{{ℜ}_{6}}>UD_{{ℜ}_{3}}$. The top three cities with the highest uncertainty are also Beijing, Tianjin, and Langfang. This is because these three cities are more complex in terms of socio-economic systems, population density, and infrastructure networks, leading to greater variability and incompleteness in the evaluation data. Consequently, decision-makers (DMs) have relatively lower confidence in the assessment results for these cities. For instance, Beijing’s multi-layered governance and rapid urbanization increase the difficulty of accurately measuring flood resilience, while Tianjin’s coastal location and industrial structure introduce additional uncertainties. Langfang, as a rapidly developing city, exhibits dynamic changes in land use and exposure, further complicating the evaluation. Combining Figure 7 and Table 3, it is shown that the ranking of flood resilience and the ranking of uncertainty exhibit a consistent order: cities with higher resilience (Beijing, Tianjin, Langfang) also show higher uncertainty, while cities with lower resilience (Baoding, Zhangjiakou, Chengde) show lower uncertainty. This positive correlation suggests that more developed and complex urban systems tend to achieve higher resilience, but at the cost of greater evaluation ambiguity. Conversely, less developed cities present more certain but poorer resilience performance. These findings highlight the need for differentiated strategies: for high-resilience yet high-uncertainty cities, further investigation to reduce epistemic uncertainty is crucial; for low-resilience cities, practical resilience enhancement measures should be prioritized.

### 6.2. Sensitivity Analysis

**Figure 8:**
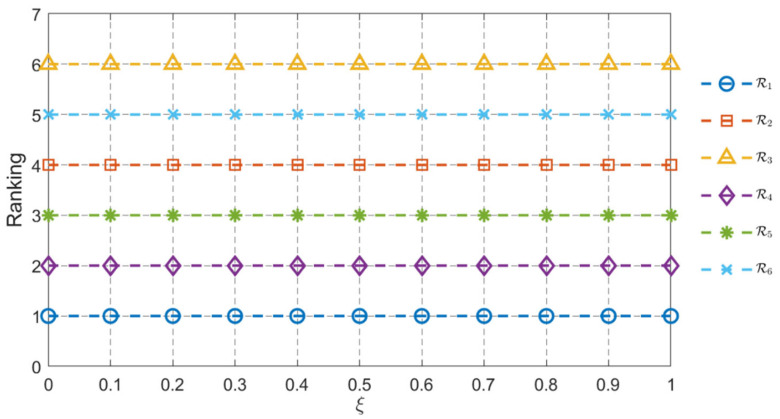
Alternative city rankings under varying balance coefficients.

The sensitivity analysis’s objective is to verify the findings and show the precision and variation in the ranking results. DMs can fine-tune the computational model and use sensitivity analysis to verify the results of the proposed method. In this section, the robustness of the ranking results is verified by changing the balancing coefficient ξ. The balance coefficient, ξ, in this case study is fixed at 0.5. The balance coefficient is progressively raised from 0 to 1 in increments of 0.1 in order to completely evaluate its sensitivity. The flood resilience levels and ranking results of each city under different balancing coefficients are shown in Table A4 and Figure 8.

Based on Table A4 and Figure 8, it can be seen that with changes in the balancing coefficient, the flood resilience levels of each city do not undergo substantial changes. Regardless of the value of the balancing coefficient, the expected evaluation score of $R_{1}$ remains consistently at 1.9993. This is because the maximum values in Q and N are $Q_{1}$ and $N_{1}$, and the expected value calculated through *Equation (24)* remains 1. $R_{2},R_{3},R_{4},R_{5}$ and $R_{6}$ gradually decrease with the increase in the balancing coefficient, but it does not affect the final ranking. In conclusion, the sensitivity to the balancing coefficient is low. Despite slight changes in the assessment values of each city’s flood resilience, the ranking results remain unchanged. The results indicate that the method exhibits good robustness.

### 6.3. Comparative Analysis

In order to verify the dependability and efficiency of the proposed method, we carried out a comparison study that included both quantitative and qualitative evaluations.

#### 6.3.1. Quantitative Analysis

In this section, we compare the results obtained using the proposed method with those from existing popular methods. The excellence and dependability of the proposed approach are proven by comparative research, giving confidence for more research and real-world applications. CRITIC-TOPSIS [24], CRITIC-VIKOR [25,26], and CV-TOPSIS [27] are employed for ranking the alternative cities. To ensure the rationality of the comparison results and avoid other influencing factors, the following assumptions are made.

(1) For the comparison of methods determining the decision-maker weights, the criteria evaluations use the mixed probability information cloud set evaluation results from Equation (26).
(2) For the comparison of methods determining the alternative city ranking, when DMs provide evaluations using precise values, the criteria evaluations use the expected values from the cloud model in Table A3 as the evaluation results.

i. Comparison with CRITIC-TOPSIS, CRITIC-VIKOR, and CV-TOPSIS

(1) Comparison with CRITIC-TOPSIS: The weights of the criteria are determined using the CRITIC method [24]. Based on the criteria evaluation results in Table A3, the computed results of all key parameters are presented in Table A5. The ranking of the alternative cities was determined by employing the TOPSIS [24], as shown in Table A6. Clearly, the ranking results of the alternative cities ${ℜ}_{1}>{ℜ}_{4}>{ℜ}_{5}>{ℜ}_{2}>{ℜ}_{6}>{ℜ}_{3}$ are consistent with those obtained by the method proposed in this paper, which demonstrates the reliability of the proposed comprehensive evaluation approach.

(2) Comparison with CRITIC-VIKOR: The weights of the criteria have been determined using the CRITIC method, as shown in Table A5. The next step involves using VIKOR [25,26] to calculate the performance of each alternative city. Based on the criteria evaluation results in Table A3, the ranking results are presented in Table A7. The ranking results of the alternative cities (${ℜ}_{4}>{ℜ}_{5}>{ℜ}_{1}>{ℜ}_{6}>{ℜ}_{2}>{ℜ}_{3}$) are largely consistent with the results obtained using the method proposed in this paper, with the main difference being that the rankings of alternative cities ${ℜ}_{1}$ and ${ℜ}_{2}$ have decreased, while the relative rankings of other alternative cities remain unchanged.

(3) Comparison with CV-TOPSIS: The criteria weights are determined using the CV method [27]. Based on Equation (A10), the computed results of all key parameters are presented in Table A8. After determining the weights, the ranking of the alternative cities was determined using the TOPSIS method [27], as shown in Table A9. The ranking results of the alternative cities ${ℜ}_{1}>{ℜ}_{4}>{ℜ}_{5}>{ℜ}_{2}>{ℜ}_{6}>{ℜ}_{3}$ are consistent with the results obtained using the method proposed in this paper, further demonstrating the reliability and effectiveness of the comprehensive evaluation method proposed in this study.

ii. Summary

**Figure 9:**
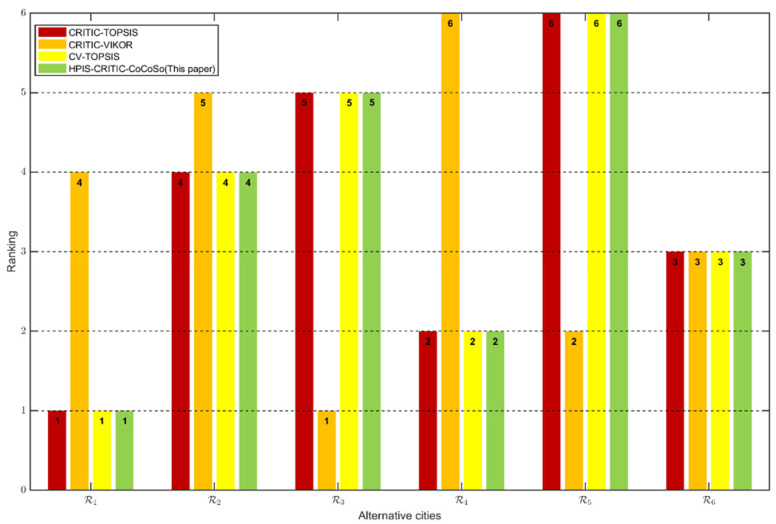
The comparison results of different methods.

The final ranking results calculated using the three multi-criteria decision-making methods—CRITIC-TOPSIS, CRITIC-VIKOR, and CV-TOPSIS—and the HPIS-CRITIC-CoCoSo method proposed in this paper are shown in Figure 9. Overall, the ranking results of the flood resilience levels of different cities are consistent, demonstrating the reliability and effectiveness of the proposed method. However, there are also subtle differences.

As shown in Figure 9 and Table 4, the ranking results of the CRITIC-TOPSIS and CV-TOPSIS methods are consistent with those of the HPIS-CRITIC-CoCoSo method, but the methods differ. Meanwhile, in the ranking results calculated by the CRITIC-VIKOR method, the rankings of ${ℜ}_{1}$ and ${ℜ}_{2}$ are both lowered, while the relative rankings of other alternative cities remain unchanged. The main advantages of this study are summarized as follows:

(1) Compared with the traditional CRITIC method, the C-CRITIC method fully integrates the cloud weight triplet and incorporates the uncertainty within the evaluation information into the weight calculation. As a result, it is well-suited for multi-criteria group decision-making problems characterized by significant uncertainty.

(2) All three methods use precise values to express the evaluation results of criteria, losing the fuzziness and randomness in the evaluation process. The proposed HPICS method in this paper preserves and propagates these uncertainties throughout the calculation process.

(3) Compared with TOPSIS and VIKOR, the C-CoCoSo technique is extended to the cloud model environment and employs the Bhattacharyya distance to precisely quantify the distance between cloud models, making it more suitable for urban flood resilience evaluation characterized by significant uncertainty.

Therefore, the method proposed in this paper offers greater flexibility in multi-criteria evaluation of complex systems. It can handle evaluations of various complex systems and provides realistic ranking results.

#### 6.3.2. Qualitative Analysis

This section mainly compares the existing Cloud-CoCoSo method to demonstrate the advantages of the approach proposed in this study.

Mandal and Khan (2022) [49] proposed the Cloud-CoCoSo method, which initially applies the traditional range method for normalization, resulting in criteria values within the range [0, 1]. This can cause issues when solving for relative scores $K_{ia},K_{ib}$ and $K_{ic}$, as the denominator may be 0 (or very close to 0), leading to distorted ranking results. In contrast, the method proposed in this paper improves the range method, ensuring that the normalized criterion falls within the range [1, 2], thus avoiding the occurrence of zero (or near-zero) denominators and providing stable ranking results. Secondly, in the original method, equal weights are assigned to all criteria, overlooking the differences in their importance. This leads to key criteria being downplayed, while the impact of less important criteria is exaggerated, distorting the evaluation results. The method presented in this paper employs an improved C-CRITIC approach, which comprehensively considers the cloud weight triplet for weight assignment. This ensures more objective and flexible evaluation results that can better handle evaluations of complex systems. Finally, a simple parameter weighting method is used to distinguish between cloud models, which is inadequate for accurately distinguishing between two membership clouds that follow a normal distribution. In this study, we use the Bhattacharyya distance to evaluate the overlap and determine the similarity between two probability distributions. This enhances the ability to differentiate between two cloud models, providing a refined measure of cloud model distinction.

### 6.4. Superiority Analysis

(1) By constructing a new evaluation information expression method, HPIS, the choice domain of evaluation information for DMs is greatly expanded, breaking the dual structural limitation of traditional linguistic term sets.
(2) The inherent uncertainty contained in the evaluation information is incorporated into the weight calculation process, resulting in a cloud weight triplet together with contrast intensity and conflict. C-CRITC is well-suited for weight determination in complex, uncertain environments.
(3) The improved CRITIC and CoCoSo methods are extended into the cloud model environment, addressing the full-process management of randomness and fuzziness in the urban flood resilience evaluation.
(4) The CoCoSo techniques are extended to the cloud model environment, where the Bhattacharyya distance is employed to precisely quantify the similarity between cloud models. This enhances the discriminative capability among the cloud models and improves the ranking performance for evaluating the flood resilience of alternative cities.
(5) A novel MCGDM framework is developed using HPIS based on the C-CRITIC and C-CoCoSo methods and is applied for the first time to the evaluation of urban flood resilience. This framework not only yields effective evaluation results but also ensures that complex uncertainty is consistently integrated throughout the entire decision-making process.

## 7. Conclusions

To overcome the limitations of MCGDM, which restrict DMs’ ability to express their judgments and result in the loss of uncertainty information, a novel MCGDM framework is proposed. On the one hand, HPIS enables DMs to provide both qualitative/quantitative evaluation information for criteria evaluations and their associated probabilities, breaking the previous dual structural limitation and significantly expanding the choice domain for DMs. On the other hand, the improved CRITIC and CoCoSo methods are extended into the cloud model environment, comprehensively considering both randomness and fuzziness in evaluation information, thus ensuring that uncertainty information is preserved throughout the entire evaluation process.

Through a case study, the proposed method is validated in the UFR evaluation process, identifying Baoding, Zhangjiakou, and Chengde as the weak links in the study area. This provides a valuable reference for enhancing the flood resilience of urban agglomerations. Sensitivity analysis shows that the ranking structure of the cities remains unchanged, with Baoding, Zhangjiakou, and Chengde consistently identified as weak links, verifying the robustness of the proposed method. The comparative analysis further verifies the effectiveness of the proposed method. Meanwhile, compared with previous methods, it demonstrates significant advantages in urban flood resilience evaluation under complex uncertainty. As with all scientific research, this work has its limitations: (1) it only considers decision-making involving a small number of DMs, without addressing large-scale group decision-making, and is therefore suitable for small expert samples; (2) it calculates DM and criteria weights solely based on objective weighting, without combining subjective weighting methods to obtain composite weights; and (3) only limited case analysis, comparative analysis, and sensitivity analysis were conducted, lacking richer external validation analysis and statistical benchmark comparisons.

## Figures and Tables

**Table 1:** Information on decision-makers.

Basic Characteristic		Percentage	Basic Characteristic		Percentage
Years of Experience	More than 5 years	50%	Professional title	Senior	50%
	3 to 5 years	25%		Associate Senior	25%
	1 to 3 years	25%		Other	25%
Occupation	Government manager	50%	Gender	Male	75%
	University teacher	50%		Female	25%

**Table 2:** Values of $\sigma^{q}$, $\eta^{q}$ and $w^{q}$.

DMs	$\sigma^{q}$	$\eta^{q}$	$w^{q}$
DM_1_	0.8216	1.3588	0.2710
DM_2_	0.7654	1.2250	0.2443
DM_3_	1.1768	1.5806	0.3153
DM_4_	0.6558	0.8490	0.1694

**Table 3:** Uncertainty measures of evaluation results.

	${ℜ}_{1}$	${ℜ}_{2}$	${ℜ}_{3}$	${ℜ}_{4}$	${ℜ}_{5}$	${ℜ}_{6}$
En+3He	0.1664	0.1302	0.1179	0.1513	0.1415	0.1248
Ranking	1	4	6	2	3	5

**Table 4:** Comparison of different methods.

Approaches	Types of Evaluation Information	Determination of Criterion Weights	Ranking Method
Mandal and Khan (2022) [49]	NCM	/	Cloud-CoCoSo
Diakoulaki et al. (1995) [34]	Exact	CRITIC	Weighted sum
Li et al. (2024) [24]	Exact	CRITIC	TOPSIS
Saraji et al. (2023) and Li et al. (2022) [25,26]	FFSs and Exact	CRITIC	VIKOR
Shan et al. (2020) [27]	Exact	CV	TOPSIS
This paper	Novel HPISs	C-CRITIC	C-CoCoSo

**Figure A1:**
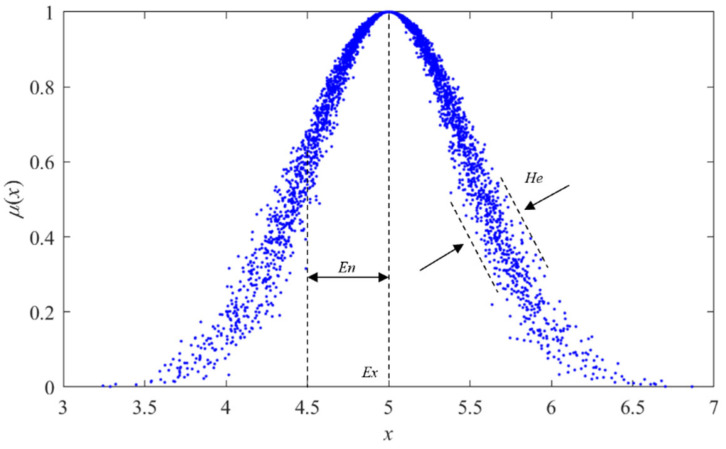
The normal cloud model plotted with parameters (5, 0.5, 0.05).

## Data Availability

The original contributions presented in this study are included in the article. Further inquiries can be directed to the corresponding author.

## Appendix A

### Appendix A.1. Preliminaries

This section reviews several key concepts and operational rules related to the normal cloud model (NCM) and PLTSs.

#### Appendix A.1.1. Normal Cloud Model

The cloud model, proposed by Academician Deyi Li (1995) [50], serves as a core model in uncertainty artificial intelligence theory, designed to address the bidirectional transformation between qualitative concepts and quantitative data. This model uniformly characterizes the fuzziness and randomness of concepts in natural language through mathematical methods, providing an innovative tool for uncertainty modeling in complex systems.

**Definition** **A1** [51]**.** *Let* x∈U *be a random instance of* C*. The discourse universe* U *defines the qualitative concept* C*. A random instance of* C *is denoted by* x∈U*. The membership degree* μ(x) *of* C *is a stochastic number exhibiting a stable trend. As shown in Equation (A1), the membership degree* μ(x) *distributed over* U *is termed the membership cloud. Each* x *is referred to as a cloud drop.*

(A1)
$$\mu :U\to [0,1]\;\forall x\in U\;x\to \mu \left(x\right)$$

Three numerical properties—expectation (*Ex*), entropy (*En*), and hyper-entropy (*He*)—are used in the cloud model to characterize an idea. *Ex* stands for the cloud droplet’s position in the universe as a mathematical average. The degree of uncertainty is measured by *En*. *He* is En’s degree of uncertainty. The normal cloud model plotted with parameters (5, 0.5, 0.05) is shown in Figure A1.

##### NCM and Its Basic Operations

The following are the definitions of the arithmetic operation rules [52,53,54,55] for NCMs $C_{1}=\left(Ex_{1},En_{1},He_{1}\right)\mathrm{and}C_{2}=\left(Ex_{2},En_{2},He_{2}\right)$ within the same universe:

(A2) $$C_{1}+C_{2}=\left(Ex_{1}+Ex_{2},\sqrt{En_{1}^{2}+En_{2}^{2}},\sqrt{He_{1}^{2}+He_{2}^{2}}\right)$$

(A3) $$C_{1}-C_{2}=\left(Ex_{1}-Ex_{2},\sqrt{En_{1}^{2}+En_{2}^{2}},\sqrt{He_{1}^{2}+He_{2}^{2}}\right)$$

(A4) $$C_{1}\times C_{2}=\left(Ex_{1}Ex_{2},\sqrt{{\left(En_{1}Ex_{2}\right)}^{2}+{\left(En_{2}Ex_{1}\right)}^{2}},\sqrt{{\left(He_{1}Ex_{2}\right)}^{2}+{\left(He_{2}Ex_{1}\right)}^{2}}\right)$$

(A5) $$T_{1}/T_{2}=\left(\frac{Ex_{1}}{Ex_{2}},\sqrt{{\left(\frac{En_{1}}{Ex_{2}}\right)}^{2}+{\left(\frac{Ex_{1}En_{2}}{Ex_{2}^{2}}\right)}^{2}},\sqrt{{\left(\frac{He_{1}}{Ex_{2}}\right)}^{2}+{\left(\frac{Ex_{1}He_{2}}{Ex_{2}^{2}}\right)}^{2}}\right)$$

**Definition** **A2** [52]**.** *Let* $C_{i}=\left(Ex_{i},En_{i},He_{i}\right)$ (i=1,2,…,n) *be a set of NCMs in* U*. The synthetic operator is a mapping CS:* $C^{n}\to C$

(A6) $$CS\left(C_{1},C_{2},\ldots ,C_{n}\right)=\left(\frac{1}{n}\sum_{i=1}^{n}Ex_{i},\frac{1}{6}\left(\underset{i}{\max}\left(Ex_{i}+3En_{i}\right)-\underset{j}{\min}\left(Ex_{j}\left.\left.\left.-3En_{j}\right)\right),\sqrt{\sum_{i=1}^{n}He_{i}^{2}}\right)\right.\right.\right.$$

**Definition** **A3** [52,53]**.** *Let* $C_{i}=\left(Ex_{i},En_{i},He_{i}\right)(i=1,2,\ldots ,n)$ *be a set of NCMs in* U*. The weighted average operator is represented by the mapping CWA:* $C^{n}\to C$

(A7) $$CWA\left(C_{1},C_{2},\ldots ,C_{n}\right)=\sum_{i=1}^{n}w_{i}C_{i}/\sum_{i=1}^{n}w_{i}$$

*where* $w_{i}$ *is the weight of* $C_{i}$.

If $w_{i}\in [0,1],i=1,2,\ldots ,n$ is a real number and $\sum_{i=1}^{n}w_{i}=1$, then Equation (A7) is simple to comprehend as follows, per the conclusion:

(A8) $$CWA\left(C_{1},C_{2},\ldots ,C_{n}\right)=\left(\sum_{i=1}^{n}w_{i}Ex_{i},\sqrt{\sum_{i=1}^{n}{\left(w_{i}En_{i}\right)}^{2}},\sqrt{\sum_{i=1}^{n}{\left(w_{i}He_{i}\right)}^{2}}\right)$$

**Definition** **A4** [53]**.** *Given two NCMs* $C_{1}=\left(Ex_{1},En_{1},He_{1}\right)$ *and* $C_{2}=\left(Ex_{2},En_{2},He_{2}\right)$. $C_{1},C_{2}\in U$*, the comparison rules are given as follows:*

(1) *If* $Ex_{1}>Ex_{2}$*, then* $C_{1}>C_{2}$*;*
(2) *If* $Ex_{1}=Ex_{2}$ *and* $En_{1}<En_{2}$*, then* $C_{1}>C_{2}$*;*
(3) *If* $Ex_{1}=Ex_{2},En_{1}=En_{2}$*, and* $He_{1}<He_{2}$*, then* $C_{1}>C_{2}$*;*
(4) *If and only if *$Ex_{1}=Ex_{2},En_{1}=En_{2}$*, and *$He_{1}=He_{2}$*, then *$C_{1}=C_{2}$*.*

##### Distance Measure of NCM

This paper employs the Bhattacharyya distance, which evaluates the similarity between two probability distributions based on their overlap, to measure the distance between NCMs. It is defined as follows:

**Definition** **A5.** *Let* $C_{1}=\left(Ex_{1},En_{1},He_{1}\right)$ *and* $C_{2}=\left(Ex_{2},En_{2},He_{2}\right)$ *be two NCMs,* $C_{1},C_{2}\in U$*, then*

$$\begin{array}{l} d\left(C_{1},C_{2}\right)=BD\left(C_{1},C_{2}\right)\\ =\frac{1}{2}\ln\frac{{\left(En_{1}\right)}^{2}+{\left(He_{1}\right)}^{2}+{\left(En_{2}\right)}^{2}+{\left(He_{2}\right)}^{2}}{2\sqrt{\left({\left(En_{1}\right)}^{2}+{\left(He_{1}\right)}^{2}\right)\left({\left(En_{2}\right)}^{2}+{\left(He_{2}\right)}^{2}\right)}}+\frac{1}{4}\frac{{\left(Ex_{1}-Ex_{2}\right)}^{2}}{{\left(En_{1}\right)}^{2}+{\left(He_{1}\right)}^{2}+{\left(En_{2}\right)}^{2}+{\left(He_{2}\right)}^{2}} \end{array}$$

#### Appendix A.1.2. Linguistic Information

Linguistic variables from LTSs are typically used by DMs in daily decision-making to convey their thoughts about the items under consideration. An LTS can be defined as:

**Definition** **A6** [15]**.** *Let* τ *be a positive integer and* $s_{\alpha }$ *be a linguistic variable. The LTS is expressed as follows:*

(A9) $$S=\left\{s_{\alpha }\left|s_{0},s_{1},\ldots ,s_{\tau }\right.\right\}$$

For the expression of multiple linguistic terms, the LEs are often derived by DMs using a comparative linguistic method as outlined by context-free grammar (*G_H_*) [56].

To represent the degree to which an object belongs to a specific linguistic evaluation term, Pang et al. [17] proposed the PLTS by integrating probability with linguistic terms. The corresponding formulation is as follows:

$$L(p)=\left\{s_{\alpha }\left(p_{\alpha }\right)|\alpha =1,2,\ldots ,\tau \right\}$$

where $s_{\alpha }\in S,p_{\alpha }\geq 0$ and $\sum_{\alpha =1}^{\tau }p_{\alpha }\leq 1$ are given. If condition $\sum_{\alpha =1}^{\tau }p_{\alpha }<1$ holds, PLTS is considered incomplete, and the remaining probability is denoted as $p_{s}=1-\sum_{\alpha =1}^{\tau }p_{\alpha }$.

#### Appendix A.1.3. Conversion of Hybrid Information

This subsection introduces the method for converting hybrid information—including exact values, interval values, linguistic terms, and linguistic expressions—into NCMs.

The method proposed by [57] for converting exact values, interval values, linguistic terms, and linguistic expressions into NCMs is as follows:

Let e be an exact value and the conversion rule $f_{E}$ is defined as follows:

(A10) e→C(e,0,0)

Let $I=[I_{l},I_{r}]$ be an interval value in the information set *S*, the conversion rule $f_{IV}$ is defined as follows.

(A11) $$I\to C\left(\frac{I_{l}+I_{r}}{2},\frac{I_{l}-I_{r}}{6},0\right)$$

DMs can express the results of their evaluations using any one of nine linguistic expressions. Following that, these language concepts are converted into their equivalent NCMs. The mapping rules $f_{LT}$ to NCMs are illustrated in Table A1.

**Table A1:** Linguistic terms encoded by NCMs.

Comment Information		Probabilistic Information	
Linguistic Terms	NCMs	Linguistic Terms	NCMs
*C*_0_: *none* (*n*)	(0.00, 0.100, 0.020)	*P*_0_: *almost impossible* (*aip*)	(0.00, 0.010, 0.002)
*C*_1_: *very low* (*vl*)	(0.197, 0.457, 0.022)	*P*_1_: *highly unlikely* (*hul*)	(0.02, 0.046, 0.002)
*C*_2_: *low*	(1.246, 1.062, 0.034)	*P*_2_: *unlikely* (*ul*)	(0.125, 0.101, 0.003)
*C*_3_: *slightly low* (*sl*)	(3.083, 1.030, 0.033)	*P*_3_: *somewhat unlikely* (*swul*)	(0.308, 0.103, 0.003)
*C*_4_: *medium* (*med*)	(5.058, 1.109, 0.056)	*P*_4_: *possible* (*p*)	(0.506, 0.111, 0.006)
*C*_5_: *slightly high* (*sh*)	(7.105, 1.027, 0.041)	*P*_5_: *somewhat likely* (*swl*)	(0.711, 0.103, 0.004)
*C*_6_: *high*	(8.743, 1.242, 0.041)	*P*_6_: *likely* (*l*)	(0.874, 0.124, 0.004)
*C_7_*: *very high* (*vh*)	(9.775, 1.054, 0.027)	*P_7_*: *highly likely* (*hl*)	(0.978, 0.105, 0.003)
*C_8_*: *maximum* (*m*)	(10.00, 0.100, 0.020)	*P_8_*: *almost certain* (*ac*)	(1.000, 0.010, 0.002)

The conversion of linguistic expressions (LEs) into an NCM is carried out in two steps ($f_{LE_{c}}$).

Step 1: Using the defined principles $f_{G_{H}}$, LEs are converted into linguistic term sets (LTSs).

(A12) $$f_{G_{H}}(s)=\left\{\begin{array}{l} \{C_{i}|C_{i}\in H\},s=C_{i}\\ \{C_{j}|C_{j}\in H{\;\mathrm{and}\;C}_{j}\leq C_{i}\},s={\mathrm{at}\;\mathrm{most}\;C}_{i}\\ \{C_{j}|C_{j}\in H{\;\mathrm{and}\;C}_{j}<C_{i}\},s={\mathrm{lower}\;\mathrm{than}\;C}_{i}\\ \{C_{j}|C_{j}\in H{\;\mathrm{and}\;C}_{j}\geq C_{i}\},s={\mathrm{at}\;\mathrm{least}\;C}_{i}\\ \{C_{j}|C_{j}\in H{\;\mathrm{and}\;C}_{j}>C_{i}\},s={\mathrm{greater}\;\mathrm{than}\;C}_{i}\\ \{C_{k}|C_{k}\in H{\;\mathrm{and}\;C}_{i}\leq C_{k}\leq C_{j}\},{\mathrm{between}\;C}_{i}{\;\mathrm{and}\;C}_{j} \end{array}\right.$$

Step 2: the LTSs are converted into an NCM using the synthetic operator provided in Definition A2 and the mapping rules $f_{LT}$.

(A13) $$LEs\overset{f_{G_{H}}}{\to }LTSs\overset{f_{LT}}{\to }NCMs\overset{CS}{\to }NCM$$

The rule $f_{LE_{p}}$ for converting probabilistic information in linguistic expressions into an NCM is the same as those used for rule $f_{LE_{c}}$. The only difference is that the comment information is replaced with probabilistic information.

### Appendix A.2. Algorithm

**Algorithm A1.** C-CRITIC for criteria weights
Step 9.1. Uncertainty (A14) $$\upsilon_{j}=\frac{\sum_{i=1}^{m}\left(En_{ij}+3He_{ij}\right)}{\sum_{j=1}^{n}\sum_{i=1}^{m}\left(En_{ij}+3He_{ij}\right)}$$ $\mathrm{where}\;\upsilon_{j}$ represents the uncertainty of the jth criterion. Step 9.2. Correlation coefficient of criteria (A15) $$\rho_{jk}=\frac{\sum_{i=1}^{m}BD\left(C_{ij},{\bar{C}}_{j}\right)BD\left(C_{ik},{\bar{C}}_{k}\right)}{\sqrt{\sum_{i=1}^{m}{\left(BD\left(C_{ij},{\bar{C}}_{j}\right)\right)}^{2}\sum_{i=1}^{m}{\left(BD\left(C_{ik},{\bar{C}}_{k}\right)\right)}^{2}}}$$ $\mathrm{where}\;{\bar{C}}_{j}$$\;\mathrm{and}\;{\bar{C}}_{k}$ represent the average values of the jth and kth criterion in different samples. They are calculated using the following formula: (A16) $${\bar{C}}_{j}=\frac{\sum_{i=1}^{m}C_{ij}}{m},\;\;\;{\bar{C}}_{k}=\frac{\sum_{i=1}^{m}C_{ik}}{m}$$ Step 9.3. Contrast intensity of each criterion $\mathrm{Let}\;\sigma_{j}$ represent criteria j’s standard deviation. (A17) $$\sigma_{j}=\sqrt{\frac{\sum_{i=1}^{m}{\left(BD\left(C_{ij},{\bar{C}}_{j}\right)\right)}^{2}}{m}}$$ Step 9.4. Criteria weights $\mathrm{Calculate}\;\mathrm{index}\;\eta_{j}$ by the following formula: (A18) $$\eta_{j}=(1-\upsilon_{j})\sigma_{j}\sum_{k=1}^{n}\left(1-\rho_{jk}\right)$$ Utilizing *Equation (A19)*, determine the weights of the criteria. (A19) $$w_{j}=\frac{\eta_{j}}{\sum_{j=1}^{n}\eta_{j}}$$

### Appendix A.3. Data

**Table A2:** DMs’ evaluation information.

DMs	Alternative City	C_1_	C_2_–C_22_	C_23_
DM_1_	${ℜ}_{1}$	**{2.5(L),3(ul),2(hul)}**	…	**{vh(0.9),m(0.1)}**
	${ℜ}_{2}$	{1.2(swl),1(swul)}		**{**at most high(0.9),m(0.1)**}**
	${ℜ}_{3}$	{2.6(0.8),3(0.1),1(0.1)}		**{**high([0.7, 0.9]),between sh and high(0.1),m(0.1)**}**
	${ℜ}_{4}$	{2(hl),3(hul)}		{high(0.8),m(0.2)}
	${ℜ}_{5}$	{2(0.8),3(0.1),1(0.1)}		{at most high(0.9),m(0.1)}
	${ℜ}_{6}$	{1.4(swl),1(ul),2(ul)}		{sh(0.6),high(0.2),med(0.2)}
DM_2_	${ℜ}_{1}$	{2.5(L),5(ul),3.5(hul)}	…	{vh(0.9),m(0.1)}
	${ℜ}_{2}$	{1.2(0.7), 2.4(0.1),1.3(0.1)}		**{**at most high(0.9),m(0.1)**}**
	${ℜ}_{3}$	{2.6(0.8),3(0.1),4.8(0.1)}		**{**high([0.7, 0.9]),at least high([0.1, 0.3])**}**
	${ℜ}_{4}$	{2(swl),3(ul),1(ul)}		{high(1.0)}
	${ℜ}_{5}$	{2(hl),4(hul)}		{at most high(0.9),m(0.1)}
	${ℜ}_{6}$	{1.4(0.8),2.1(0.1),0.9(0.1)}		{sh(0.6),high(0.2),m(0.2)}
DM_3_	${ℜ}_{1}$	{2.5([0.6, 0.7]),3([0.1, 0.2]),2(0.1)}	…	{vh(0.9),high(0.05),m(0.05)}
	${ℜ}_{2}$	{1.2(between swl and l),1.6(hul),0.7(ul)}		**{**at most high(hl),vh(between aip and hul)**}**
	${ℜ}_{3}$	{2.6(0.9),3(0.1) }		**{**high([0.7, 0.9]),sh(0.05),vh(0.15)**}**
	${ℜ}_{4}$	{1.4(0.6),1.6(0.4)}		{high(l),sh(ul),vh(between hul and ul),m(between aip and hul)}
	${ℜ}_{5}$	{2(0.6),2.2(0.2),2.1(0.2)}		{at most high(l), vh(ul),m(hul)}
	${ℜ}_{6}$	{2(0.8),2.8(0.1),1.5(0.1)}		{sh(hl),sl(hul),med(hul), between sh and high(hul) }
DM_4_	${ℜ}_{1}$	{2.5([0.65, 0.8]),2.7([0.2, 0.3])}	…	{vh(hl),between high and vh(hul)}
	${ℜ}_{2}$	{1.2(0.5),1.6(0.3),0.7(0.2)}		**{**at most high(hl), between high and vh (hul)**}**
	${ℜ}_{3}$	{2.6(0.4),3(0.3),2.2(0.3)}		**{**high([0.7, 0.9]),between sh and high([0.1, 0.3])**}**
	${ℜ}_{4}$	{2(0.7),2.2(ul),2.1(0.15)}		{high(ac)}
	${ℜ}_{5}$	{1.4(0.8),1.6(0.1),1.5(0.1)}		{at most high(hl), between sh and high (hul)}
	${ℜ}_{6}$	{2(0.8),2.5(0.2)}		{sh(p),high(swul),med(ul)}

**Note:** Due to limited space for displaying the table data, ‘…’ is used to indicate that this part of the data has been omitted.

**Table A3:** Criteria evaluation and weights.

Criteria	${ℜ}_{1}$	${ℜ}_{2}$	${ℜ}_{3}-{ℜ}_{5}$	${ℜ}_{6}$	$w_{j}$	Rank
C_1_	(0.0350,0.1914,0.0061)	(1.0000,0.1832,0.0062)	**…**	(0.6379,0.1577,0.0051)	0.0778	3
C_2_	(1.0000,0.4735,0.0178)	(0.1705,0.2916,0.0103)	…	(0.5549,0.3364,0.0131)	0.0116	20
C_3_	(0.0336,0.4691,0.0170)	(0.0872,0.4456,0.0151)	**…**	(0.2963,0.4652,0.0161)	0.0139	19
C_4_	(1.0000,0.2517,0.0081)	(0.2402,0.2260,0.0084)	…	(0.6631,0.2258,0.0072)	0.0283	14
C_5_	(0.7765,0.6988,0.0240)	(0.2968,0.6530,0.0224)	**…**	(0.0177,0.6689,0.0238)	0.0061	23
C_6_	(0.0000,0.2428,0.0081)	(1.0000,0.3096,0.0112)	…	(0.6876,0.2698,0.0092)	0.0199	17
C_7_	(1.0000,0.4378,0.0169)	(0.2157,0.2359,0.0084)	**…**	(0.2121,0.1067,0.0042)	0.1452	2
C_8_	(0.6179,0.2095,0.0084)	(0.0000,0.1930,0.0072)	…	(0.0390,0.1872,0.0075)	0.0411	11
C_9_	(0.8694,0.2202,0.0082)	(0.2768,0.1440,0.0053)	**…**	(0.1296,0.1239,0.0043)	0.0560	5
C_10_	(0.8013,0.5651,0.0215)	(1.0000,0.6051,0.0237)	…	(0.7585,0.5174,0.0203)	0.0081	21
C_11_	(0.2517,0.7882,0.0334)	(0.0000,0.7439,0.0276)	**…**	(0.3077,0.7273,0.0284)	0.0075	22
C_12_	(1.0000,0.3223,0.0129)	(0.4776,0.1995,0.0072)	…	(0.2703,0.1467,0.0055)	0.1494	1
C_13_	(1.0000,0.2545,0.0097)	(0.0000,0.1594,0.0072)	**…**	(0.2240,0.1764,0.0078)	0.0419	10
C_14_	(1.0000,0.4582,0.0149)	(0.0385,0.3216,0.0109)	…	(0.1052,0.2631,0.0092)	0.0142	18
C_15_	(1.0000,0.0000,0.0000)	(0.0000,0.0000,0.0000)	**…**	(0.1150,0.0000,0.0000)	0.0270	15
C_16_	(1.0000,0.3176,0.0123)	(0.0160,0.1664,0.0078)	…	(0.0700,0.1663,0.0076)	0.0472	9
C_17_	(1.0000,0.2291,0.0095)	(0.5228,0.1804,0.0078)	**…**	(0.0000,0.1601,0.0073)	0.0605	4
C_18_	(1.0000,0.3143,0.0109)	(0.0000,0.0956,0.0036)	…	(0.1053,0.1292,0.0044)	0.0499	6
C_19_	(1.0000,0.3684,0.0119)	(0.3224,0.2409,0.0081)	**…**	(0.2687,0.1553,0.0054)	0.0362	13
C_20_	(0.9776,0.2445,0.0101)	(0.3520,0.1632,0.0073)	…	(0.0548,0.1711,0.0076)	0.0479	7
C_21_	(1.0000,0.2727,0.0106)	(0.0000,0.2045,0.0090)	**…**	(0.3719,0.2116,0.0091)	0.0384	12
C_22_	(0.0000,0.2457,0.0108)	(0.8308,0.2661,0.0113)	…	(0.7644,0.2537,0.0108)	0.0475	8
C_23_	(1.0000,0.3202,0.0125)	(0.0007,0.2913,0.0119)	**…**	(0.5780,0.2660,0.0107)	0.0244	16

**Note:** Due to limited space for displaying the table data, ‘…’ is used to indicate that this part of the data has been omitted.

**Table A4:** The evaluation results of the alternative cities under different parameters.

ξ	${ℜ}_{1}$	${ℜ}_{2}$	${ℜ}_{3}-{ℜ}_{4}$	${ℜ}_{5}$	${ℜ}_{6}$
0.0	(1.9993,0.1490,0.0058)	(1.7944,0.1169,0.0045)	**…**	(1.8710,0.1268,0.0049)	(1.7709,0.1121,0.0044)
0.1	(1.9993,0.1489,0.0058)	(1.7931,0.1169,0.0045)	**…**	(1.8702,0.1268,0.0049)	(1.7695,0.1120,0.0043)
0.2	(1.9993,0.1489,0.0058)	(1.7916,0.1168,0.0045)	**…**	(1.8692,0.1267,0.0049)	(1.7678,0.1119,0.0043)
0.3	(1.9993,0.1489,0.0058)	(1.7897,0.1167,0.0045)	**…**	(1.8680,0.1267,0.0049)	(1.7657,0.1119,0.0043)
0.4	(1.9993,0.1489,0.0058)	(1.7872,0.1167,0.0045)	**…**	(1.8663,0.1267,0.0049)	(1.7629,0.1118,0.0043)
0.5	(1.9993,0.1490,0.0058)	(1.7838,0.1167,0.0045)	**…**	(1.8642,0.1267,0.0049)	(1.7592,0.1118,0.0043)
0.6	(1.9993,0.1494,0.0058)	(1.7791,0.1168,0.0045)	**…**	(1.8611,0.1269,0.0049)	(1.7539,0.1119,0.0043)
0.7	(1.9993,0.1502,0.0058)	(1.7719,0.1172,0.0045)	**…**	(1.8565,0.1274,0.0050)	(1.7459,0.1123,0.0044)
0.8	(1.9993,0.1524,0.0059)	(1.7595,0.1187,0.0046)	**…**	(1.8485,0.1292,0.0050)	(1.7321,0.1137,0.0044)
0.9	(1.9993,0.1604,0.0062)	(1.7334,0.1247,0.0048)	**…**	(1.8318,0.1359,0.0053)	(1.7031,0.1195,0.0046)
1.0	(1.9993,0.2093,0.0081)	(1.6422,0.1637,0.0064)	**…**	(1.7742,0.1779,0.0069)	(1.6009,0.1570,0.0061)

**Note:** Due to limited space for displaying the table data, ‘…’ is used to indicate that this part of the data has been omitted.

**Table A5:** Values of $\sigma_{j}$, $\eta_{j}$ and $w_{j}$.

Criteria	$\sigma_{j}$	$\eta_{j}$	$w_{j}$
C_1_	0.3831	1.6496	0.0413
C_2_	0.3759	1.2188	0.0305
C_3_	0.3861	2.9115	0.0728
C_4_	0.3545	1.4940	0.0374
C_5_	0.3360	1.5569	0.0389
C_6_	0.3603	1.5556	0.0389
C_7_	0.3464	1.4626	0.0366
C_8_	0.3324	1.8337	0.0459
C_9_	0.3801	1.4563	0.0364
C_10_	0.4031	1.5964	0.0399
C_11_	0.3925	2.3972	0.0600
C_12_	0.3676	1.7508	0.0438
C_13_	0.3449	1.5316	0.0383
C_14_	0.3569	1.7589	0.0440
C_15_	0.4244	1.9100	0.0478
C_16_	0.4047	1.8288	0.0457
C_17_	0.4010	2.1187	0.0530
C_18_	0.3944	1.0495	0.0263
C_19_	0.3308	1.8527	0.0463
C_20_	0.4217	1.7957	0.0449
C_21_	0.3579	1.4147	0.0354
C_22_	0.3567	1.7559	0.0439
C_23_	0.4303	2.0801	0.0520

**Note:** The criteria parameter values $\eta_{j}$, criteria weights $w_{j}$ and the standard deviation $\sigma_{j}$.

**Table A6:** Based on the final ranking and related parameters of TOPSIS (weights come from CV).

Alternative City	$D^{+}$	$D^{-}$	$\hbar_{i}$	Ranking
${ℜ}_{1}$	0.0827	0.1211	0.5943	1
${ℜ}_{2}$	0.1233	0.0608	0.3302	4
${ℜ}_{3}$	0.1243	0.0515	0.2930	6
${ℜ}_{4}$	0.0757	0.1050	0.5810	2
${ℜ}_{5}$	0.0823	0.0962	0.5390	3
${ℜ}_{6}$	0.1123	0.0547	0.3278	5

**Note:** The positive ideal solution $D^{+}$, the negative ideal solution $D^{-}$ and the ranking results $\hbar_{i}$ of each alternative city.

**Table A7:** Based on the final ranking and related parameters of VIKOR.

Alternative City	$S_{i}$	$R_{i}$	$Q_{i}$	Ranking
${ℜ}_{1}$	0.2551	0.0711	0.4671	3
${ℜ}_{2}$	0.7134	0.0671	0.8613	5
${ℜ}_{3}$	0.7441	0.0728	1.0000	6
${ℜ}_{4}$	0.3402	0.0463	0.0870	1
${ℜ}_{5}$	0.4800	0.0498	0.2954	2
${ℜ}_{6}$	0.6963	0.0530	0.5767	4

**Note:** The maximum group utility $S_{i}$, the minimum individual regret $R_{i}$, and the comprehensive evaluation index $Q_{i}$.

**Table A8:** Based on the CV criteria weights and related parameters.

Criteria	${\bar{\mathbb{C}}}_{j}$	$\sigma_{j}$	$\delta_{j}$	$w_{j}$
C_1_	0.4573	0.4573	0.8378	0.0428
C_2_	0.4310	0.4310	0.8720	0.0445
C_3_	0.3287	0.3831	1.1749	0.0600
C_4_	0.4142	0.3759	0.8559	0.0437
C_5_	0.3735	0.3861	0.8997	0.0460
C_6_	0.4219	0.3545	0.8538	0.0436
C_7_	0.3956	0.3360	0.8756	0.0447
C_8_	0.4412	0.3603	0.7534	0.0385
C_9_	0.4027	0.3464	0.9441	0.0482
C_10_	0.4940	0.3324	0.8161	0.0417
C_11_	0.4424	0.3801	0.8872	0.0453
C_12_	0.5368	0.4031	0.6848	0.0350
C_13_	0.3424	0.3925	1.0075	0.0515
C_14_	0.3310	0.3676	1.0785	0.0551
C_15_	0.5772	0.3449	0.7353	0.0376
C_16_	0.5039	0.3569	0.8032	0.0410
C_17_	0.4981	0.4244	0.8052	0.0411
C_18_	0.3954	0.4047	0.9974	0.0509
C_19_	0.3874	0.4010	0.8538	0.0436
C_20_	0.4759	0.3944	0.8861	0.0453
C_21_	0.5341	0.3308	0.6700	0.0342
C_22_	0.7100	0.4217	0.5024	0.0257
C_23_	0.5491	0.3579	0.7836	0.0400

**Note:** The average value ${\bar{\mathbb{C}}}_{j}$ and standard deviation $\sigma_{j}$ of the criterion, the coefficient of variation $\delta_{j}$, and criteria weights $w_{j}$.

**Table A9:** Based on the final ranking and related parameters of TOPSIS (weights come from CRITIC).

Alternative City	$D^{+}$	$D^{-}$	$\hbar_{i}$	Ranking
${ℜ}_{1}$	0.0720	0.1320	0.6470	1
${ℜ}_{2}$	0.1264	0.0608	0.3247	4
${ℜ}_{3}$	0.1302	0.0442	0.2533	5
${ℜ}_{4}$	0.0843	0.0975	0.5363	2
${ℜ}_{5}$	0.0907	0.0867	0.4885	6
${ℜ}_{6}$	0.1157	0.0545	0.3202	3

**Note:** The positive ideal solution $D^{+}$, the negative ideal solution $D^{-}$ and the ranking results $\hbar_{i}$ of each alternative city.
